# ChIP-chip versus ChIP-seq: Lessons for experimental design and data analysis

**DOI:** 10.1186/1471-2164-12-134

**Published:** 2011-02-28

**Authors:** Joshua WK Ho, Eric Bishop, Peter V Karchenko, Nicolas Nègre, Kevin P White, Peter J Park

**Affiliations:** 1 Department of Medicine, Brigham and Women's Hospital, and Harvard Medical School, Boston, MA, USA; 2 Center for Biomedical Informatics, Harvard Medical School, Boston, MA, USA; 3 Program in Bioinformatics, Boston University, Boston, MA, USA; 4 Institute for Genomics and Systems Biology, University of Chicago, Chicago, IL, USA; 5 Informatics Program, Children's Hospital, Boston, MA, USA

## Abstract

**Background:**

Chromatin immunoprecipitation (ChIP) followed by microarray hybridization (ChIP-chip) or high-throughput sequencing (ChIP-seq) allows genome-wide discovery of protein-DNA interactions such as transcription factor bindings and histone modifications. Previous reports only compared a small number of profiles, and little has been done to compare histone modification profiles generated by the two technologies or to assess the impact of input DNA libraries in ChIP-seq analysis. Here, we performed a systematic analysis of a modENCODE dataset consisting of 31 pairs of ChIP-chip/ChIP-seq profiles of the coactivator CBP, RNA polymerase II (RNA PolII), and six histone modifications across four developmental stages of *Drosophila melanogaster*.

**Results:**

Both technologies produce highly reproducible profiles within each platform, ChIP-seq generally produces profiles with a better signal-to-noise ratio, and allows detection of more peaks and narrower peaks. The set of peaks identified by the two technologies can be significantly different, but the extent to which they differ varies depending on the factor and the analysis algorithm. Importantly, we found that there is a significant variation among multiple sequencing profiles of input DNA libraries and that this variation most likely arises from both differences in experimental condition and sequencing depth. We further show that using an inappropriate input DNA profile can impact the average signal profiles around genomic features and peak calling results, highlighting the importance of having high quality input DNA data for normalization in ChIP-seq analysis.

**Conclusions:**

Our findings highlight the biases present in each of the platforms, show the variability that can arise from both technology and analysis methods, and emphasize the importance of obtaining high quality and deeply sequenced input DNA libraries for ChIP-seq analysis.

## Background

Chromatin immunoprecipitation (ChIP) followed by genomic tiling microarray hybridization (ChIP-chip) or massively parallel sequencing (ChIP-seq) are two of the most widely used approaches for genome-wide identification and characterization of *in vivo *protein-DNA interactions. They can be used to analyze many important DNA-interacting proteins including RNA polymerases, transcription factors, transcriptional co-factors, and histone proteins [1]. Indeed these genome-wide ChIP analysis approaches have led to many important discoveries related to transcriptional regulation [2-4], epigenetic regulation through histone modification [5], nucleosome organization [6,7], and interindividual variation in protein-DNA interactions [8,9].

ChIP-chip first appeared in the literature about 10 years ago and was one of the earliest approaches to performing genome-wide mapping of protein-DNA interactions in organisms with small genomes, such as yeast [2,10]. Currently, various tiling microarray platforms of common model organisms are well supported by commercial vendors, and many bioinformatics tools have been developed for ChIP-chip analysis [11-14]. Fueled by rapid development of the second generation high-throughput sequencing technologies in the past few years, ChIP-seq has emerged as an attractive alternative to ChIP-chip [1]. For instance, ChIP-seq generally produces profiles with higher spatial resolution, dynamic range, and genomic coverage, allowing it to have higher sensitivity and specificity over ChIP-chip in terms of protein binding site identification. Further, ChIP-seq can be used to analyze virtually any species with a sequenced genome since it is not constrained by the availability of an organism-specific microarray. Many current ChIP-seq protocols can work with a smaller amount of initial material compared to ChIP-chip [15,16]. Moreover, ChIP-seq is already a more cost-effective way of analyzing mammalian genomes, and the cost effectiveness will likely become more apparent as the cost of high-throughput sequencing technology continues to drop. These factors have led to the rapid adoption of ChIP-seq technology.

However, despite the widespread use of both ChIP-chip and ChIP-seq, only a few small-scale studies have attempted to quantitatively compare these technologies using real data. Euskirchen et al. [17] compared the STAT1 binding sites identified by ChIP-chip and ChIP-PET (paired-end ditag sequencing by Sanger sequencing technology) and found that there was a good overall agreement between the two technologies, particularly at identifying highly ranked enrichment regions. They nonetheless noted specific discrepancies in regions associated with repetitive elements, which can be attributed to lack of microarray probe coverage or misalignment of ChIP-PET reads. More recently, a number of studies compared genome-wide transcription factor binding datasets generated from ChIP-chip with those generated from ChIP-seq [18-22] (see Additional file 1: Table S1). The general conclusions from these studies were that binding profiles generated from ChIP-chip and ChIP-seq were largely correlated at the genome-wide level, and that ChIP-seq had superior sensitivity and specificity over ChIP-chip in terms of binding site identification as determined by motif enrichment or quantitative PCR validation. It was also found that the strongest peaks were more likely to be detected by both technologies. However, only a few pairs of ChIP-chip/ChIP-seq profiles were analyzed in these studies, and their focus was mainly on the ability to identify narrow enrichment regions using specific peak calling algorithms. As shown previously [23] and in this study, peak identification can be strongly dependent on the analysis algorithm, so other more general comparison metrics should be used.

In addition, little is known about the technology-specific variation for analyzing histone modification data. ChIP-based histone modification data is commonly used to reconstruct average signal profiles, or "epigenetic signatures," of key genomic regions such as the transcription start and end sites, but the impact of using ChIP-chip versus ChIP-seq data for constructing epigenetic signatures is largely unknown. Furthermore, it is also important to understand technology-specific biases associated with high-throughput sequencing. Recent studies indicated that the distribution of cross-linked and sonicated DNA fragments (input DNA) was affected by chromatin structure, copy number variation, occurrence of genomic repeats, mappability, genomic location, gene expression activity, and genomic GC content variation [24-26]. Since input DNA is commonly used as a background control for a ChIP-seq experiment, it is important to assess how such variation affects the analysis of ChIP-seq data.

Therefore a thorough understanding of the technological variation between ChIP-chip and ChIP-seq is important in experimental design and data analysis. In this study, we compiled and analyzed 31 pairs of ChIP-chip/ChIP-seq profiles of technical replicates across eight immunoprecipitation (IP) factors (CBP, RNA PolII, and six histone modifications) at four developmental stages of the common fruit fly *Drosophila melanogaster *(Table 1) as part of the model organism Encyclopedia of DNA Elements (modENCODE) project [27]. In addition, our compiled dataset comprises another 62 ChIP-chip profiles (biological replicates) in the same set of biological conditions (i.e., three ChIP-chip biological replicates at each developmental stage/IP combination), nine sequencing profiles of input DNA, and four pairs of ChIP-seq/ChIP-seq replicates (Table 2). Agilent's tiling microarray (Agilent custom 3X244K Dmel Whole Genome Tiling Microarray) and Illumina's Genome Analyzer II platforms were used to generate the ChIP-chip and ChIP-seq data, respectively. All data used in this study were generated as part of the modENCODE project, and are accessible from NCBI GEO (accession numbers: GSE15292, GSE16013, and GSE20000). The goal of this study was to quantify reproducibility within and between profiles generated using ChIP-chip and ChIP-seq approaches, and to pinpoint the source of variation between the technologies, which ultimately should provide useful information for experimental design and data analysis.

**Table 1:** Summary of the ChIP-chip and ChIP-seq profiles analyzed in this study

ChIP-chip and ChIP-seq replicates								
**IP factor**	**E-0-4 h**		**E-12-16 h**		**E-16-20 h**		**E-20-24 h**	
	**chip**	**seq**	**chip**	**seq**	**chip**	**seq**	**chip**	**seq**

**CBP**	3	1	---	---	3	1	3	1
**H3K27Ac**	3	1	3	1	3	1	3	1
**H3K27Me3**	3	1	3	1	3	1	3	1
**H3K4Me1**	3	1	3	1	3	1	3	1
**H3K4Me3**	3	1	3	1	3	1	3	1
**H3K9Ac**	3	1	3	1	3	1	3	1
**H3K9Me3**	3	1	3	1	3	1	3	1
**PolII**	3	1	3	1	3	1	3	1
**INPUT**	*	1	*	1	*	1	*	1

For each combination of developmental stage and IP factor, three biological samples were profiled by ChIP-chip, and one of these samples was independently profiled by ChIP-seq. Therefore we have three biological ChIP-chip/ChIP-chip replicates, and one technical ChIP-chip/ChIP-seq replicate pair for every biological condition. *Although no independent microarray profiles of input DNA were available, we extracted input DNA profiles from the input channel (of the two-channel microarray) for each ChIP-chip profile. We therefore have 31 × 3 = 93 INPUT-chip profiles in this collection, where all INPUT-chip profiles within each time point are technical replicates.

**Table 2:** Summary of the additional ChIP-seq profiles analyzed in this study

ChIP-seq replicates only				
**IP factor**	**E-4-8 h**	**AdultMale**	**AdultFemale**	**S2 cell**
	
	**seq**	**seq**	**seq**	**seq**

**CBP**	2	2	2	---
**CTCF**	---	---	---	2
**INPUT**	1	1	1	2

Details of the four additional pairs of ChIP-seq/ChIP-seq (biological) replicates analyzed in this study. The two CTCF ChIP-seq profiles were technical replicates, whereas all other ChIP-seq pairs were biological replicates.

## Results

### Analysis of input DNA profiles

To understand the technological differences between sequencing-based and microarray-based ChIP data, we first analyzed the profiles of cross-linked and sonicated DNA fragments (input DNA) generated by microarray (INPUT-chip) and high-throughput sequencing (INPUT-seq). Since the input DNA profile should be independent of the antibody used for ChIP, this comparison can give insight into the specific differences between these two profiling technologies. We obtained INPUT-chip data from the background channel of our two-channel microarray data. While this microarray platform uses competitive hybridization, the two channels in our Agilent microarray have been shown to be relatively independent as saturation in either channel is very rare [28]. Out of all the INPUT-chip profiles that we extracted, we only present the analysis of eight representative profiles here (two from each of the four developmental time points) since most of the INPUT-chip profiles are very similar (Additional file 2: Figure S1). The eight INPUT-chip profiles were then compared with the nine INPUT-seq profiles collected in this study (Additional file 1: Table S3).

**Figure 1:**
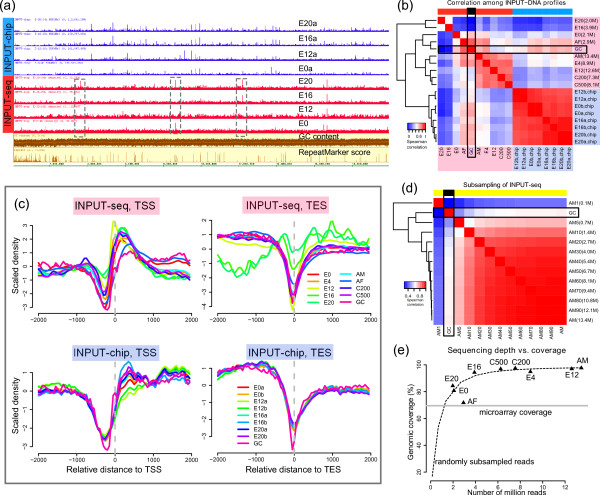
**Comparison of input DNA profiles obtained by microarray and sequencing technologies**. (a) A genome browser view of input DNA profiles of chromosome 2R of *D. melanogaster *at various developmental stages measured by microarray (INPUT-chip; blue) and sequencing (INPUT-seq; red). (b) A heat map that summarizes the Spearman correlation coefficient between every pair of the nine INPUT-seq and eight INPUT-chip profiles along with genome-wide GC content. The number of mappable reads (in million) is written next to the name of each INPUT-seq profile. (c) The average signal profiles of INPUT-seq and INPUT-chip around transcription start sites (TSSs) and transcription end sites (TESs) are largely consistent, and their variation along these genomic regions generally coincide with GC content variation. (d) We generated 11 additional profiles from one of the INPUT-seq samples (AM) by subsampling the reads at different proportions (90%,80%,...,10%,5%,1%). A heat map summary representation of the Spearman correlation coefficient between every pair of sub-sampled INPUT-seq profiles and GC content is shown here. (e) The relationship between sequencing depth and genomic coverage. The curve shows how sequence read subsampling (i.e., reducing sequencing depth) affects genomic coverage. The genomic coverage of the nine INPUT-seq datasets and our Agilent microarray are also shown in the plot.

One of the most striking observations is that INPUT-chip and INPUT-seq profiles appear to be substantially different, even though the same input DNA material was used for microarray hybridization and sequencing (Figure 1). The relative magnitude and location of the peaks seem to be consistent across the INPUT-chip profiles from multiple experiments. However, the patterns in the nine INPUT-seq profiles appear to be more variable. We can visually identify many regions that have inconsistent signal enrichment across multiple INPUT-seq profiles (highlighted in Figure 1a). A clustering analysis was performed to quantify this observation. We found that all eight INPUT-chip profiles clustered closely to one another (Figure 1b). This result shows that the background DNA distribution measured from microarray and high-throughput sequencing is different. All INPUT-chip and seven out of nine INPUT-seq profiles correlated positively with genomic GC content at the genome-wide level (Figure 1b), as well as around the transcription start sites (TSS) and transcription end sites (TES) (Figure 1c). The strength of the correlation with GC is highly consistent among INPUT-chip profiles, but highly variable among the INPUT-seq profiles (Figure 1b-c and Additional file 2: Figure S2). Notably, the INPUT-seq profiles obtained at E-16-20 h (E16) and E-20-24 h (E20) do not correlate with GC content.

We also note that INPUT-seq with higher sequencing depth (>4 million mapped reads) tend to cluster together more tightly than those with lower sequencing depth, suggesting that there may be a relationship between sequencing depth and input DNA variability. To test this hypothesis, we generated 11 additional INPUT-seq profiles by subsampling sequencing reads from the most deeply sequenced input DNA sample (AdultMale; AM) at different sampling proportion (Figure 1d and Additional file 2: Figure S3). As expected, profiles with higher sequencing depth tend to cluster more strongly together, and their correlation with GC content variation is more consistent. However, the GC content correlation only becomes much weaker only at a very low sequencing depth (<2 million reads; Figure 1d). This indicates that low sequencing depth is not the only factor affecting INPUT-seq quality. Moreover, some INPUT-seq with relatively low sequencing depth (E0 and AF, <4 million reads) can give consistent input DNA profiles. This implies that INPUT-seq variability may also be attributed to other experimental factors. Although further studies are required to dissect the full range of experimental factors affecting variability of input DNA libraries, it could be affected by variations in the sample preparation (e.g., different chromatin preparation and sonication), run-to-run variation of the sequencer, sequencer-to-sequencer variation for the same model, and a host of other variables in experiments. The high variability among INPUT-seq profiles is indeed a critical issue, since large variability contributes to instability of density estimation in a ChIP-seq profile, which will affect downstream data analysis. As will be shown in subsequent sections of this paper, an INPUT-seq with unusually weak correlation with GC content can impact construction of average profiles at important genomic locations. It is thus imperative to sequence the input DNA to sufficient depth and to ascertain that the obtained profile is consistent with those from similar experiments.

Genomic coverage is another key consideration when choosing between ChIP-chip and ChIP-seq. The genomic coverage of ChIP-chip is limited by the microarray probe design, and the coverage of ChIP-seq is dependent on sequencing depth. The genomic coverage achieved by our Agilent microarray is about 70%. Using the sub-sampled INPUT-seq data, we show that INPUT-seq generally provides higher genomic coverage at sequencing depth as low as one million reads. This trend constructed from the randomly subsampled data corroborates the observed genomic coverage of the other eight real INPUT-seq datasets (Figure 1e).

### Comparison of profile characteristics

**Figure 2:**
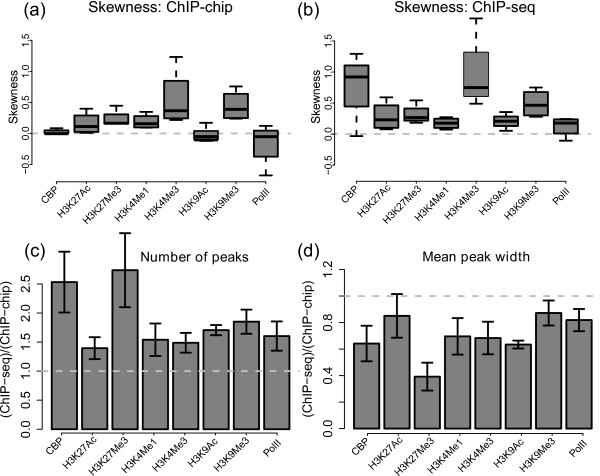
**Comparison of the characteristics of ChIP-chip and ChIP-seq profiles**. Figures (a) and (b) summarize the skewness of the signal distributions of all the ChIP-chip and ChIP-seq profiles. A ChIP profile with a good signal-to-noise ratio should have a signal distribution that is positively skewed (i.e., skewness >0). Higher skewness implies a better signal-to-noise ratio. In almost all cases, a ChIP-seq profile has higher signal skewness than its corresponding ChIP-chip profile. Figures (c) and (d) show the ratios of the number and mean width of the enrichment regions identified by ChIP-chip and ChIP-seq using our heuristic approach (see the Methods section of this paper). In almost all cases, we can identify larger number and narrower peaks in a ChIP-seq profile than its corresponding ChIP-chip profile.

We then compared the characteristics of ChIP-chip and ChIP-seq profiles. To compare the profiles generated by the two technologies, we divided the genome into 1 kb non-overlapping bins and defined the enrichment level at each bin as the average of log ratio of the IP channel over the input channel (see the Methods section for details). We refer to a signal distribution of a ChIP profile as its distribution of enrichment values of all the bins. First, we aimed to characterize the average signal-to-noise ratio for profiles generated by the two technologies. We used the (truncated) skewness of the signal density profile after removing signals from the highest and lowest 5% of the distribution as a measure of signal-to-noise ratio of a profile. Skewness is a measure of asymmetry of a distribution and a positive skewness indicates that the tail on the right side is longer, implying a good signal-to-noise ratio. In almost all cases, a ChIP-seq profile has a higher skewness than its corresponding ChIP-chip profile of the same biological condition (Figure 2 and Additional file 1: Table S4). We note that the difference of skewness is dependent on the IP factor which could be due to different antibody quality and prevalence of histone modification or binding events. The same conclusion can be drawn even if a different bin size was used (Additional file 2: Figure S4). Our results confirmed the general observation that ChIP-seq usually produces a more distinctive signal profile than ChIP-chip.

Next, we characterized the enrichment regions within each ChIP profile. To perform a fair comparison, we would like to use an algorithm that performs peak calling on ChIP-seq and ChIP-chip data using the same criteria. Currently, many commonly used peak calling algorithms are specifically designed for analyzing ChIP-chip or ChIP-seq data, but not both. To overcome this limitation, we identified peaks from both ChIP-chip and ChIP-seq profiles using the same genome-scanning heuristic (see the Methods section). Our results indicate that we can almost always discover a larger number of peaks and narrower peaks using data generated from ChIP-seq compared to ChIP-chip when analyzing the same biological sample, and this conclusion is consistent regardless of the stringency of the identification criteria used (Figure 2 and Additional file 2: Figure S5). In practice, we can probably identify an even larger number of narrow peaks in ChIP-seq data if we explicitly make use of strand-specific information within the peak calling procedure (beside only shifting each read towards its 5' end by a constant number of base pair), so the current analysis provides a lower bound on the effectiveness of ChIP-seq compared to ChIP-chip. Taken together, our results demonstrate that ChIP-seq provides higher spatial resolution and signal-to-noise ratio.

### Genome-wide signal reproducibility within and between technologies

**Figure 3:**
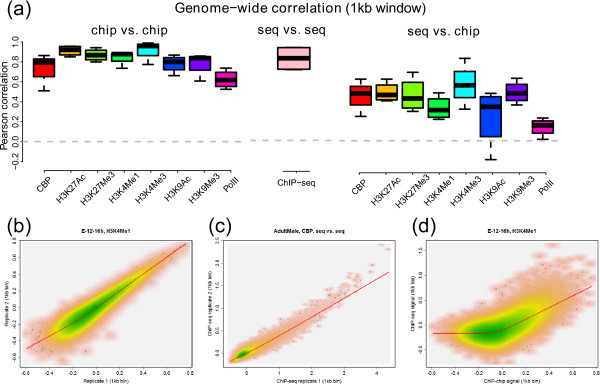
**Genome-wide reproducibility within and between ChIP-chip and ChIP-seq replicate profiles**. (a) Genome-wide reproducibility between two profiles was measured by Pearson's correlation coefficient, *r*, between their signal intensities (in 1 kb bins). Both ChIP-chip and ChIP-seq have high reproducibility (median *r*≈0.83), whereas the reproducibility between replicate ChIP-chip and ChIP-seq profiles is moderate (median *r*≈0.41). This figure also shows one typical example of genome-wide correlation between biological replicates of (b) ChIP-chip and ChIP-chip, (c) ChIP-seq and ChIP-seq, and (d) ChIP-chip and ChIP-seq. A locally weighted scatterplot smoothing (LOESS) regression line is also shown in each of these plots.

Further, we estimated the reproducibility between ChIP-chip and/or ChIP-seq profiles at the genome-wide level (1 kb bins). To avoid biases due to differences in genomic coverage and sequence mapping (Figure 1e), we exclude genomic regions that do not contain any microarray probes and regions with unusually high variability across multiple INPUT-seq profiles. The Pearson correlation coefficient, *r*, was used as a measure of correlation, since it is more sensitive than the Spearman correlation coefficient for comparing the tail of two signal distributions, which is particularly important in analyzing ChIP enrichment signal profiles. The correlation between ChIP-chip replicate pairs and between ChIP-seq replicate pairs is generally high (median *r *= 0.85 and 0.82, respectively), indicating that both technologies can produce reproducible results. As expected, the cross-platform correlation between replicate pairs of ChIP-chip and ChIP-seq profiles are more modest (median *r *= 0.41; Additional file 1: Table S5). Similar conclusions can be reached even if we use different bin sizes for calculating inter-profile correlation (Additional file 2: Figure S6). A representative scatter plot comparing each pair of technologies is shown in Figure 3b-d. We also observe a positive correlation between the skewness and inter-profile reproducibility (Additional file 2: Figure S7), suggesting more sensitive antibodies may produce more consistent profiles between the two technologies.

### Construction of average signal profile at TSS and TES

**Figure 4:**
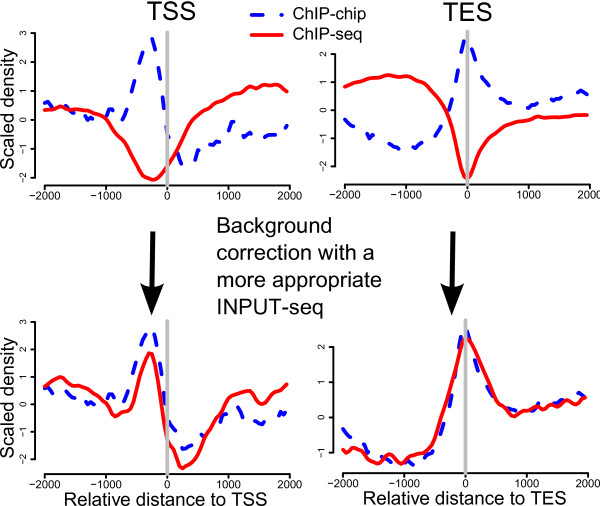
**Illustration of how variability in an INPUT-seq profile can affect reconstruction of average signal profile at TSS and TES**. The top panel shows the average signal profiles at the TSS and TES for the ChIP-chip and ChIP-seq profiles of H3K27Me3 at E-16-20 h. These ChIP-chip and ChIP-seq profiles differ quite substantially, and the ChIP-seq profiles resemble that of the GC content variation (Figure 1c). We subsequently reprocessed the ChIP-seq sample by using the INPUT-seq at AdultFemale as background for normalization since this profile has a strong correlation with GC content variation, which more likely reflect the actual technology-specific biases of our sequencing platform. After this procedure, the average signal profiles of ChIP-chip and ChIP-seq look much more alike, indicating that the original INPUT-seq at E-16-20 h does not appropriately capture the technology-specific variation at these sites.

Construction of average ChIP signal profiles around important genomic features such as TSS and TES is a common way to visualize signal enrichment around these features. Therefore, we investigated the reproducibility of average TSS and TES profiles (2 kb up and 2 kb downstream) for every pair of replicate ChIP profiles (Additional file 2: Figure S8). The average profiles of most replicate pairs are highly consistent. However, there are a few pairs that are significantly different, especially the profiles of H3K27Me3 and H3K9Me3 at both stage E-16-20 h and E-20-24 h (Additional file 2: Figures S8c and S8g). Without external validation, it is impossible to determine whether the average signal profiles generated by ChIP-chip or ChIP-seq are more accurate. Nonetheless, two lines of evidence led us to believe that the average signal profiles from ChIP-chip were more likely to be accurate. First, all three ChIP-chip replicates at these time points had very consistent average profiles. Second, the ChIP-seq average signal profiles at these biological conditions resembled the trend of GC content variation at TSS and TES (Figure 1c). The unusually low correlations between GC contents and the INPUT-seq profiles of E-16-20 h and E-20-24 h (Figure 1b and Additional file 2: Figure S2b) prompted us to hypothesize that the observed discrepancy was due to a misrepresentation of GC content variation by the respective INPUT-seq profiles. Both H3K27Me3 and H3K9Me3 are repressive marks that are usually depleted at TSSs and TESs so any variation in background subtraction is likely much more pronounced than other histone marks that have strong signal enrichment at these genomic features. To test our hypothesis, we replaced the corresponding INPUT-seq background with the INPUT-seq from the AdultFemale sample, since it has the highest correlation with GC content variation. After the replacement, the average signal profiles generated by ChIP-seq and ChIP-chip at these two developmental stages agree (Figure 4 and Additional file 2: Figure S9). This result is striking since it shows that using different INPUT-seq as negative control of the same ChIP-seq profile can lead to substantially different interpretation of the data.

### Effect of using different input profiles in ChIP-seq data normalization

**Figure 5:**
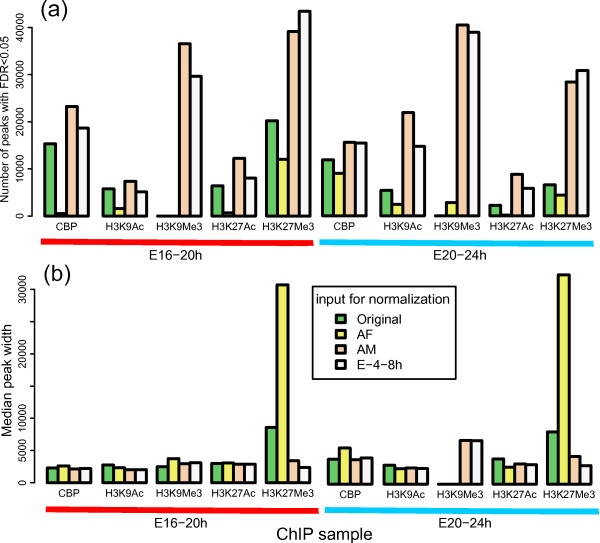
**Effect of normalization with different INPUT-seq on ChIP-seq peak calling**. We compared the number of peaks (a) and median peak width (b) of 10 ChIP-seq samples (CBP, H3K9Ac, H3K9Me3, H3K27Ac, H3K27Me3 at E16-20 h and E20-24 h) where each of them was normalization against four different input DNA samples (the input for from the matching time point, AdultFemale, AdultMale, and E-4-8 h). Peak calling was performed with SPP using the same parameters. Clearly peak detection is significantly affected by using different input DNA library as background control. In general, more peaks are identified as statistically significant (FDR < 0.05) when normalized with an INPUT-seq library with higher sequencing depth, although the magnitude of the differences vary across different ChIP datasets.

Having observed the impact of INPUT-seq in constructing average TSS and TES profiles, we asked whether using different INPUT-seq profiles for background normalization significantly affects ChIP-seq peak calling results. We used SPP [29] to call peaks for 10 of our ChIP-seq samples (CBP, H3K9Ac, H3K9Me3, H3K27Ac, H3K27Me3 at E16-20 h and E20-24 h) where each ChIP profiles was normalized against four different INPUT-seq as background (the input from the matching time point, AdultFemale, AdultMale, and E-4-8 h). These INPUT-seq profiles were chosen because they have different sequencing depth and correlation with GC content (Figure 1b). A comparison of the number of peaks and median peak width is shown in Figure 5. We observed a large difference in number of peaks being called for any ChIP-seq sample when different INPUT-seq was used as background. In the extreme case (E-16-24 h, H3K9Me3 ChIP), the number of peaks can changes from zero to nearly 40,000 at a FDR of 5% (Figure 5a). In general, more statistically significant peaks (FDR < 0.05) were detected when normalizing against a deeply sequenced input DNA sample (AdultMale and E-4-8 h in this experiment), although the absolute magnitude of the difference varies among ChIP datasets. The difference in peak number likely indicates a difference in detection power. For each ChIP sample, we calculated the proportion of overlap between each pair of peak sets generated by four different input DNA background (ie, six comparisons per ChIP sample). We found that the mean proportion of overlap with respect to the smaller peak set is about 95%, indicating that the differences in number of detected peak is likely due to different power to call weaker peaks. We observed that the strong peaks (ie, those with low detection FDR) were more likely detected in different peak sets (see Additional file 2: Figure S10 for an example). The median width of the detected peaks is also affected by using different INPUT-seq as background (Figure 5b). This analysis showed that the normalization using different INPUT-seq may have a significant, and underappreciated, impact on peak calling.

### Assessing variation due to the use of different peak callers

**Figure 6:**
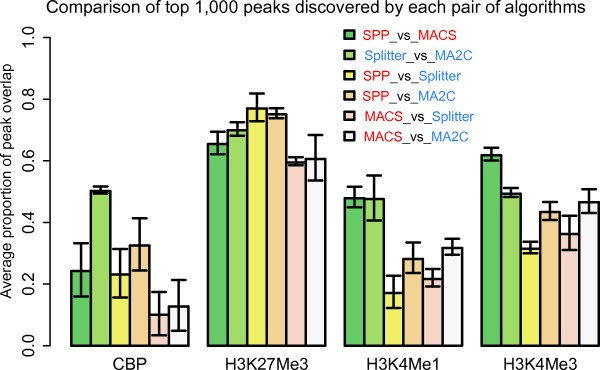
**Variability due to peak calling algorithms**. We compared the average proportion of overlapping peaks identified by two ChIP-seq peak callers (red) and two ChIP-chip peak callers (blue) for the ChIP-seq and ChIP-chip profiles, respectively. Interestingly, the variation in peak identification concordance due to the use of different algorithms can be as large as technological differences, which is especially clear in the comparison of CBP and H3K27Me3 profiles.

Another important source of variation in analysis of ChIP-chip and ChIP-seq profiles originates from the use of different analysis algorithms. A large number of publicly available ChIP-chip and ChIP-seq analysis tools have been developed to date [23,30], and all of them utilize different methods for tag shifting, profile normalization, smoothing, peak identification, and calculation of false discovery rate. It is therefore not too surprising to find that different peak callers can generate quite different results in terms of binding site identification, particularly when dealing with peaks with weak signals [23,31]. Using our compendium of ChIP-chip and ChIP-seq datasets, we could assess how much variation in peak identification can be attributed to the use of different profiling technology and use of different peak callers. In this study, we analyzed our ChIP-chip profiles using two peak callers: MA2C [13] and Splitter [32] and analyzed our ChIP-seq profiles using another two peak callers: MACS [20] and SPP [29] (see Additional file 1: Table S8). These peak callers were chosen because they are widely used, publicly available, and generally show good performance in previous comparative studies [30,31]. We calculated the overlap of the top 1,000 peaks of four of the factors (CBP, H3K4Me1, H3K4Me3, and H3K27Me3) across multiple developmental stages. The four IP factors were chosen as they were representative profiles containing broad peaks (CBP and H3K27Me3) and narrow peaks (H3K4Me1 and H3K4Me3). Here, we only present the results of comparing the top 1,000 peaks, since this is a biologically reasonable number of high-confidence enrichment sites in these profiles. The general conclusion of this analysis is robust against a variety of peak calling thresholds (Additional file 2: Figure S11). Concordance between two peak sets was measured by the average proportion of overlapping peaks. As shown in Figure 6, the comparisons based on profiles of H3K4Me1 and H3K4Me3 yielded expected results, in which the intra-platform concordance is higher than cross-platform concordance (i.e., peak sets generated by two peak callers on the same profile are more concordant than peak sets generated by two peak callers on two profiles). However, the intra-platform concordance can be as low as the inter-platform concordance when analyzing the profiles of H3K27Me3 and CBP, implying that variation in peak calling algorithms can be as large as the use of different profiling technologies for some IP factors. The observation that current peak calling algorithms produce less concordant results for ChIP profiles with broad domains (CBP and H3K27Me3) than those with sharp peaks (H3K4Me1 and H3K4Me3) may suggest that they are less consistent at identifying broad enrichment regions, which may be an interesting subject for further investigation.

## Discussion

ChIP-seq is an attractive alternative to ChIP-chip due to its many practical advantages. However, to date there is a lack of systematic comparison between ChIP-chip and ChIP-seq based on a large dataset from multiple IP factors. Using a compilation of replicate ChIP-chip and ChIP-seq datasets generated as part of the modENCODE project, we had an unprecedented opportunity to conduct such a systematic comparison. Through comparing the characteristics of the profiles generated by ChIP-chip and ChIP-seq, we showed that ChIP-seq generates profiles with higher signal-to-noise ratios and a larger number of more localized peaks. This is consistent with many previous observations that ChIP-seq generates profiles with higher spatial resolution and dynamic range. Not surprisingly, we found that inter-technology (i.e., ChIP-chip vs. ChIP-seq) reproducibility was lower than intra-technology reproducibility (ChIP-chip vs. ChIP-chip or ChIP-seq vs. ChIP-seq). We only had access to four ChIP-seq/ChIP-seq replicate profiles across two IP factors (CBP and CTCF) in this study, so the estimate of intra-platform reproducibility of ChIP-seq may be less accurate than that of ChIP-chip. Nonetheless, the overall magnitude of intra-platform reproducibility should still be instructive.

Another important lesson was that variability due to the use of different peak callers can be as substantial as different profiling technologies. Only a small number of representative peak callers were selected in this study, since it was not our primary goal to compare performance of different algorithms. Instead, our goal was to estimate to what extent peak detection variability might be attributed to the use of different profiling technologies (microarray or sequencing) and use of different peak calling algorithms. The observation that a higher variability in peak detection is associated with broad enrichment domains is interesting, and it requires further assessment using larger numbers of peak callers.

Perhaps one of the most striking findings is that there is high variability among different INPUT-seq compared to INPUT-chip. The differences between INPUT-seq profiles do not seem to correlate with the developmental stages, but rather to sequencing depth (Figure 1b and Additional file 2: Figure S3). Nonetheless, sequencing depth alone does not account for all the observed variability. Samples E0, E16, E20 and AF all have low sequencing depth (< 4 million mapped reads), yet AF and E0 have strong correlation with GC content, whereas E20 and E16 have little correlation with GC content (Figure 1b and Additional file 2: Figure S3). This suggests that variation in experimental conditions may also contribute to such variability. Our results demonstrate that it is important for the input DNA background to capture technology-specific biases, such as GC content variation, as this may have impact on the construction of average signal profiles at important genomic sites. Such average signal profiles have been used as features for building computational models of transcription factor binding or regulatory elements [33,34], so it is important to ensure that the average signal profiles are accurate. In addition, we also show that the quality of input DNA profile used for background normalization when calling peaks for a ChIP-seq dataset is critical (Figure 5). The observation that more peaks were being called when a ChIP-seq dataset was normalized against a more deeply sequenced INPUT-seq suggests that increasing the sequencing depth in the INPUT-seq data may lead to higher statistical power in ChIP-seq peak detection. Currently the importance of input DNA in ChIP-seq analysis is largely underappreciated and most researchers do not even bother to check for the consistency of the input profiles. This work provides quantitative evidence that the success of a ChIP-seq analysis may depend on the quality of input library as much as the quality of ChIP library. Since using an appropriate input DNA profile as background is critical, we believe that obtaining high quality and deeply sequenced input DNA profile is necessary, and that INPUT-seq quality should be assessed more carefully during data analysis.

We recognize there are several limitations in our study. First, we only compared ChIP-chip profiles generated from one commercial platform (Agilent tiling microarray) with ChIP-seq profiles generated from one sequencing platform (Illumina GAII). Although strictly speaking we can only make conclusion about these platforms, we believe that the key lessons learned in this study are instructive for analyzing data generated from other platforms. We note that the Agilent platform uses long oligonucleotides as probes (50-60 mers) and has relatively low noise level compared to other platforms [17,31]; Illumina's GAII sequencing platform is currently the most widely used platform for ChIP-seq, so our analysis should be of interest to many users. Second, unlike previous comparative studies using transcription factor binding data (Additional file 1: Table S1) or "spike in" experiments [31], we do not have external validation of the true enrichment regions, which prohibited us from assessing detection sensitivity and specificity. Nonetheless, we believe that our conclusions, drawn from analyzing many pairs of replicate ChIP-chip/seq profiles, should be reasonably robust.

## Conclusions

Our findings highlight the differences between ChIP-chip and ChIP-seq, and show the variability that can arise from both technology and analysis methods. We demonstrate the importance of obtaining high quality and deeply sequenced input DNA libraries for ChIP-seq analysis, which has fundamental implication to experimental design and data analysis.

## Methods

### ChIP-chip and ChIP-seq protocols

Chromatin immunoprecipitations have been performed as described previously [35]. Briefly, the biological material is homogenized in the presence of 1.8% formaldehyde. The cross-linked chromatin is sonicated using a Bioruptor (Diagenode) to an average size of 500 bp. Pre-cleared chromatin extract is incubated overnight at 4°C with the specific antibody and immunoprecipitated with protein-A Sepharose beads. After purification of the DNA and amplification of the libraries by linker-mediated PCR, the samples are labeled by incorporating Cy3 or Cy5 conjugated dUTPs. Labeled IP and Input samples are hybridized onto the Agilent 1X244K arrays using a TecanHS4800Pro at 67°C for 24 h. Details related to this custom microarray platform can be found in Additional file 1: Table S9. The non amplified ChIP samples have been used directly for sequencing by Solexa Genome Analyzer following Illumina protocols for libraries generation, cluster generation and sequencing.

### Data preprocessing

For each set of Agilent microarray data, the processed intensity value of the IP and input channels were extracted from the raw data file. For each lane of Illumina sequencing data, the raw sequence reads (36 bp single end) were extracted. Bowtie [36] was used to map both the microarray probe sequences and the Illumina's short reads onto the reference *D. melanogaster *genome assembly (dm3, FlyBase built 5.22). This procedure ensured that there was no systematic bias due to mapping to different version of the genome assembly. All microarray probe sequences can be uniquely mapped to the genome, and the proportion of mappable reads in our ChIP-seq data is available in Additional file 1: Table S2. For preprocessing of ChIP-seq data, the mapped reads of an IP library and its corresponding input DNA libraries from the same developmental stage were processed by an R package called SPP [29]. In particular, we used SPP to filter out uninformative reads, remove read anomaly and estimate the average fragment length by the cross-correlation profile (Additional file 1: Table S7). We then divided the genome into many 50 bp non-overlapping bins, and each bin *i *is associated with a genomic coordinate *x_i _*(corresponding to the center of the bin) and an intensity value *y_i _*An enrichment value in a bin is the log ratio of the smoothed signal intensity (or smoothed read count for ChIP-seq) of the IP sample over the INPUT sample. We used a Gaussian smoother with a bandwidth of 50 for signal smoothing. Specifically, the Gaussian smoother takes the form

$$y_{i}=\sum_{j}\exp\left[\frac{{\left(x{'}_{j}-x_{i}\right)}^{2}}{50}\right]y{'}_{j}$$

where *x*'*_j _*and *y*'*_j _*are the center of the genomic coordinate and intensity value of probe *j *in the same chromosome. In practice due to computation constraints, we only consider the 400 closest probes from bin *i *(200 probes upstream and 200 probes downstream) for smoothing which already give a close approximation to the full Gaussian smoother. Profiles at this 50 bp resolution were used for construction of average profiles of TSS and TES. For other analyses presented in this study, we combined the enrichment value of every 20 adjacent 50 bp bins (by averaging) to obtain a ChIP profile at the 1 kb resolution. To avoid biases in estimation of genome-wide correlation between two profiles, we first excluded genomic regions that did not contain microarray probes and had high variability in INPUT-seq variability. INPUT-chip and INPUT-seq profiles were obtained in a similar manner, except we used the log_2 _(intensity from input profile) as a measure of enrichment value.

Unless specified otherwise, all data analysis in this study was performed using the R statistical programming environment [37]. All the signal density profiles were visualized using the Integrated Genome Browser [38].

### Construction of average signal profile at TSS and TES

We used the gene model annotation from FlyBase [39] to define transcription start and end sites. We only included genes with a minimum length of 2 kb (7,231 of 15,186 genes) to exclude short genes in our analysis. The 80 bins (50 bp each) surrounding every relevant genomic feature (corresponding to 2 kb upstream and 2 kb downstream of the feature) were taken and averaged. The resulting average profiles were scaled such that the mean and the variance of signal in each profile were zero and one, respectively.

### Characterization of enrichment signals in ChIP-chip and ChIP-seq profiles

We calculated skewness of a signal density profile using the following formula:

$$skewness(x_{1},x_{2},...,x_{n})=\frac{\frac{1}{n}\sum_{i=1}^{n}{\left(x_{i}-\bar{x}\right)}^{3}}{{\left[\frac{1}{n}{\sum_{i=1}^{n}\left(x_{i}-\bar{x}\right)}^{2}\right]}^{3/2}}$$

where *x_i _*is the enrichment value of bin *i *of a genome. We specifically removed bins with enrichment value at the lowest or the highest 5% of the distribution to remove potential outliers.

We devised a simple heuristic method to detect "peaks" that can be identified by ChIP-chip and ChIP-seq profiles in a consistent manner. This method consists of two steps: (1) identify candidate enrichment regions using *ad hoc *criteria, and (2) assign a *p*-value to each candidate enrichment region. For step 1, we identify all bins with an enrichment value above $\bar{x}+ks$, where $\bar{x}$ and *s *are, respectively, the sample mean and standard deviation of a signal density per chromosome, and *k *is an arbitrary parameter. We present the results for *k *= 0 in this paper, but we have also performed analysis with *k *= -1 and *k *= 4, and the conclusion of the analyses is largely similar (Additional file 2: Figure S5). Adjacent bins are merged to form candidate enrichment regions. For step 2, we assign a statistical significance, *p*, to each enrichment region to be *p *= *P*(*l *>*L*), where *l *is the sum of enrichment values of all the bins in the candidate enrichment region, $L∼N(m\bar{x},ms^{2})$, and *m *is the number of bins in this enrichment region. To account for multiple comparison, we calculated a false discovery rate (FDR) value for each enrichment region using the method of Benjamini and Hochberg [40], and all the enrichment regions with an FDR less than 0.05 were considered statistically significant. We then characterize each set of enrichment regions from a profile by its number and median width of the enrichment regions.

## Competing interests

The authors declare that they have no competing interests.

## Authors' contributions

JWKH designed and performed the study, and wrote the manuscript. EB and PVK performed initial data analysis. NN and KPW generated the ChIP-chip and ChIP-seq datasets. PJP conceived, designed, and supervised the study. All authors read and approved the final manuscript.

## Supplementary Material

Additional file 1 **Supplemental tables**. This file contains supplementary tables. Click here for file

Additional file 2 **Supplemental figures**. This file contains supplementary figures. Click here for file

## References

[B1] Park PJ ChIP-seq: advantages and challenges of a maturing technology Nat Rev Genet 2009 10 669 680 10.1038/nrg2641 19736561 PMC3191340

[B2] Lee TI Rinaldi NJ Robert F Odom DT Bar-Joseph Z Gerber GK Hannett NM Harbison CT Thompson CM Simon I Transcriptional Regulatory Networks in Saccharomyces cerevisiae Science 2002 298 799 804 10.1126/science.1075090 12399584

[B3] Chen X Xu H Yuan P Fang F Huss M Vega VB Wong E Orlov YL Zhang W Jiang J Integration of external signaling pathways with the core transcriptional network in embryonic stem cells Cell 2008 133 1106 1117 10.1016/j.cell.2008.04.043 18555785

[B4] Nielsen R Pedersen TÅ Hagenbeek D Moulos P Siersbæk R Megens E Denissov S Børgesen M Francoijs K-J Mandrup S Genome-wide profiling of PPARγ:RXR and RNA polymerase II occupancy reveals temporal activation of distinct metabolic pathways and changes in RXR dimer composition during adipogenesis Genes Dev 2008 22 2953 2967 10.1101/gad.501108 18981474 PMC2577787

[B5] Barski A Cuddapah S Cui K Roh T Schones D Wang Z Wei G Chepelev I Zhao K High-Resolution Profiling of Histone Methylations in the Human Genome Cell 2007 129 823 837 10.1016/j.cell.2007.05.009 17512414

[B6] Heintzman ND Hon GC Hawkins RD Kheradpour P Stark A Harp LF Ye Z Lee LK Stuart RK Ching CW Histone modifications at human enhancers reflect global cell-type-specific gene expression Nature 2009 459 108 112 10.1038/nature07829 19295514 PMC2910248

[B7] Tolstorukov MY Kharchenko PV Goldman JA Kingston RE Park PJ Comparative analysis of H2A.Z nucleosome organization in the human and yeast genomes Genome Res 2009 19 967 977 10.1101/gr.084830.108 19246569 PMC2694475

[B8] Kasowski M Grubert F Heffelfinger C Hariharan M Asabere A Waszak SM Habegger L Rozowsky J Shi M Urban AE Variation in Transcription Factor Binding Among Humans Science 2010 328 232 235 10.1126/science.1183621 20299548 PMC2938768

[B9] McDaniell R Lee B-K Song L Liu Z Boyle AP Erdos MR Scott LJ Morken MA Kucera KS Battenhouse A Heritable Individual-Specific and Allele-Specific Chromatin Signatures in Humans Science 2010 328 235 239 10.1126/science.1184655 20299549 PMC2929018

[B10] Ren B Robert F Wyrick JJ Aparicio O Jennings EG Simon I Zeitlinger J Schreiber J Hannett N Kanin E Genome-Wide Location and Function of DNA Binding Proteins Science 2000 290 2306 2309 10.1126/science.290.5500.2306 11125145

[B11] Johnson WE Li W Meyer CA Gottardo R Carroll JS Brown M Liu XS Model-based analysis of tiling-arrays for ChIP-chip Procs Natl Acad Sci USA 2006 103 12457 12462 10.1073/pnas.0601180103 PMC1567901 16895995

[B12] Qi Y Rolfe A MacIsaac KD Gerber GK Pokholok D Zeitlinger J Danford T Dowell RD Fraenkel E Jaakkola TS High-resolution computational models of genome binding events Nat Biotech 2006 24 963 970 10.1038/nbt1233 16900145

[B13] Song J Johnson WE Zhu X Zhang X Li W Manrai A Liu J Chen R Liu XS Model-based analysis of two-color arrays (MA2C) Genome Biol 2007 8 R178 R178 10.1186/gb-2007-8-8-r178 17727723 PMC2375008

[B14] Droit A Cheung C Gottardo R rMAT-- an R/Bioconductor package for analyzing ChIP-chip experiments Bioinformatics 2010 26 678 679 10.1093/bioinformatics/btq023 20089513

[B15] Adli M Zhu J Bernstein BE Genome-wide chromatin maps derived from limited numbers of hematopoietic progenitors Nat Meth 2010 7 615 618 10.1038/nmeth.1478 PMC2924612 20622861

[B16] Goren A Ozsolak F Shoresh N Ku M Adli M Hart C Gymrek M Zuk O Regev A Milos PM Chromatin profiling by directly sequencing small quantities of immunoprecipitated DNA Nat Meth 2010 7 47 49 10.1038/nmeth.1404 PMC2862482 19946276

[B17] Euskirchen GM Rozowsky JS Wei C-L Lee WH Zhang ZD Hartman S Emanuelsson O Stolc V Weissman S Gerstein MB Mapping of transcription factor binding regions in mammalian cells by ChIP: Comparison of array- and sequencing-based technologies Genome Res 2007 17 898 909 10.1101/gr.5583007 17568005 PMC1891348

[B18] Ji H Jiang H Ma W Johnson DS Myers RM Wong WH An integrated software system for analyzing ChIP-chip and ChIP-seq data Nat Biotech 2008 26 1293 1300 10.1038/nbt.1505 PMC2596672 18978777

[B19] Rozowsky J Euskirchen G Auerbach RK Zhang ZD Gibson T Bjornson R Carriero N Snyder M Gerstein MB PeakSeq enables systematic scoring of ChIP-seq experiments relative to controls Nat Biotech 2009 27 66 75 10.1038/nbt.1518 PMC2924752 19122651

[B20] Zhang Y Liu T Meyer C Eeckhoute J Johnson D Bernstein B Nussbaum C Myers R Brown M Li W Model-based Analysis of ChIP-Seq (MACS) Genome Biol 2008 9 R137 R137 10.1186/gb-2008-9-9-r137 18798982 PMC2592715

[B21] Robertson G Hirst M Bainbridge M Bilenky M Zhao Y Zeng T Euskirchen G Bernier B Varhol R Delaney A Genome-wide profiles of STAT1 DNA association using chromatin immunoprecipitation and massively parallel sequencing Nat Methods 2007 4 651 657 10.1038/nmeth1068 17558387

[B22] Qin ZS Yu J Shen J Maher CA Hu M Kalyana-Sundaram S Yu J Chinnaiyan AM HPeak: an HMM-based algorithm for defining read-enriched regions in ChIP-seq data BMC Bioinformatics 2010 11 369 10.1186/1471-2105-11-369 20598134 PMC2912305

[B23] Laajala T Raghav S Tuomela S Lahesmaa R Aittokallio T Elo L A practical comparison of methods for detecting transcription factor binding sites in ChIP-seq experiments BMC Genomics 2009 10 618 618 10.1186/1471-2164-10-618 20017957 PMC2804666

[B24] Teytelman L Özaydin B Zill O Lefrançois P Snyder M Rine J Eisen MB Impact of Chromatin Structures on DNA Processing for Genomic Analyses PLoS ONE 2009 4 e6700 e6700 10.1371/journal.pone.0006700 19693276 PMC2725323

[B25] Vega VB Cheung E Palanisamy N Sung W-K Inherent Signals in Sequencing-Based Chromatin-ImmunoPrecipitation Control Libraries PLoS ONE 2009 4 e5241 e5241 10.1371/journal.pone.0005241 19367334 PMC2666154

[B26] Auerbach RK Euskirchen G Rozowsky J Lamarre-Vincent N Moqtaderi Z Lefrancois P Struhl K Gerstein M Snyder M Mapping accessible chromatin regions using Sono-Seq Proc Natl Acad Sci USA 2009 106 14926 14931 10.1073/pnas.0905443106 19706456 PMC2736440

[B27] Celniker SE Dillon LAL Gerstein MB Gunsalus KC Henikoff S Karpen GH Kellis M Lai EC Lieb JD MacAlpine DM Unlocking the secrets of the genome Nature 2009 459 927 930 10.1038/459927a 19536255 PMC2843545

[B28] Zahurak M Parmigiani G Yu W Scharpf R Berman D Schaeffer E Shabbeer S Cope L Pre-processing Agilent microarray data BMC Bioinformatics 2007 8 142 142 10.1186/1471-2105-8-142 17472750 PMC1876252

[B29] Kharchenko PV Tolstorukov MY Park PJ Design and analysis of ChIP-seq experiments for DNA-binding proteins Nat Biotech 2008 26 1351 1359 10.1038/nbt.1508 PMC2597701 19029915

[B30] Wilbanks EG Facciotti MT Evaluation of Algorithm Performance in ChIP-Seq Peak Detection PLoS ONE 2010 5 e11471 e11471 10.1371/journal.pone.0011471 20628599 PMC2900203

[B31] Johnson DS Li W Gordon DB Bhattacharjee A Curry B Ghosh J Brizuela L Carroll JS Brown M Flicek P Systematic evaluation of variability in ChIP-chip experiments using predefined DNA targets Genome Res 2008 18 393 403 10.1101/gr.7080508 18258921 PMC2259103

[B32] Splitter http://zlab.bu.edu/splitter

[B33] Won K-J Ren B Wang W Genome-wide prediction of transcription factor binding sites using an integrated model Genome Biol 2010 11 R7 R7 10.1186/gb-2010-11-1-r7 20096096 PMC2847719

[B34] Firpi HA Ucar D Tan K Discover Regulatory DNA Elements Using Chromatin Signatures and Artificial Neural Network Bioinformatics 2010 26 1579 1586 10.1093/bioinformatics/btq248 20453004 PMC2887052

[B35] Negre N Lavrov S Hennetin J Bellis M Cavalli G Mapping the distribution of chromatin proteins by ChIP on chip Methods Enzymol 2006 410 316 341 10.1016/S0076-6879(06)10015-4 16938558

[B36] Langmead B Trapnell C Pop M Salzberg S Ultrafast and memory-efficient alignment of short DNA sequences to the human genome Genome Biol 2009 10 R25 R25 10.1186/gb-2009-10-3-r25 19261174 PMC2690996

[B37] R: A language and environment for statistical computing http://www.R-project.org

[B38] Nicol JW Helt GA Blanchard SG Raja A Loraine AE The Integrated Genome Browser: free software for distribution and exploration of genome-scale datasets Bioinformatics 2009 25 2730 2731 10.1093/bioinformatics/btp472 19654113 PMC2759552

[B39] Drysdale RA Crosby MA The FlyBase C FlyBase: genes and gene models Nucl Acids Res 2005 33 D390-395-D390-395 15608223 10.1093/nar/gki046 PMC540000

[B40] Benjamini Y Hochberg Y Controlling the False Discovery Rate: A Practical and Powerful Approach to Multiple Testing J R Stat Soc Ser B 1995 57 289 300

