# Supplementary material for: ChIP-chip versus ChIP-seq: Lessons for experimental design and data analysis
Source: BMC Genomics. 2011 Feb 28;12:134. doi: 10.1186/1471-2164-12-134 (PMC3053263; doi:10.1186/1471-2164-12-134)
Supplement: Additional file 1 — Supplemental tables. This file contains supplementary tables. [file 1471-2164-12-134-S1.PDF]

Supplemental Tables for "ChIP-chip versus ChIP-seq: Lessons for experimental design and data analysis"

Joshua W.K. Ho, Eric Bishop, Peter V. Kharchenko, Nicolas Nègre, Kevin P. White, Peter J. Park

Table S1. A summary of the key findings in previous studies on ChIP-chip/ChIP-seq comparisons.

Studies	Findings
**IP:** NRSF<br>**Evaluation:** motif occurrence<br>**Peak caller:** CisGenome<br>**Ref:** (Ji et al. 2008)	1. Clear global correlation<br>2. Peaks in ChIP-chip are wider<br>3. ChIP-seq peaks are more likely to contains conserved NRSF motifs, and therefore more likely to be true positives
**IP:** PolII and STAT1<br>**Evaluation:** Comparison with ChIP-PCR of selected genomic regions.<br>**Peak caller:** PeakSeq<br>**Ref:** (Rozowsky et al. 2009)	1. ChIP-seq generates fewer false positives<br>2. ChIP-seq generates more peaks<br>3. ChIP-seq peaks are generally closer to the binding site motif<br>4. Findings consistent with (Robertson et al. 2007)
IP:** FOXA1*<br>Evaluation:** Motif occurrence*<br>***Peak caller:** MACS*<br>***Ref:** (Zhang et al. 2008)*	1. ChIP-chip has more binding sites at 1% FDR<br>2. Many false negative in ChIP-chip is due to lack of microarray probes.<br>3. ChIP-chip peaks with higher enrichment are more likely to be discovered by ChIP-seq.<br>4. Average peak width in ChIP-chip is twice of that of ChIP-seq peaks.<br>5. ChIP-seq peaks are localized to the sequence motif.

Table S2. Summary of the mappability of the raw sequencing data.

Sample	IP factor	number of reads generated	number of mapped reads	% mapped	IP/INPUT
**E-0-4h**	CBP	21,613,653	10,111,380	46.78	4.88
	K27Ac	6,681,957	4,630,953	69.31	2.23
	K27Me3	905,444	654,088	72.24	0.32
	K4Me1	4,588,861	2,652,346	57.8	1.28
	K4Me3	12,010,867	8,552,639	71.21	4.13
	K9Ac	6,753,165	4,466,744	66.14	2.15
	K9Me3	6,755,125	4,764,026	70.52	2.3
	PolII	6,456,889	340,471	5.27	0.16
	INPUT	6,699,750	2,073,217	30.94	1
**E-12-16h**	K27Ac	10,135,164	8,278,482	81.68	0.66
	K27Me3	6,910,712	5,525,926	79.96	0.44
	K4Me1	10,218,224	7,396,998	72.39	0.59
	K4Me3	10,079,436	7,075,734	70.2	0.56
	K9Ac	9,601,185	6,941,337	72.3	0.55
	K9Me3	9,854,049	8,264,618	83.87	0.66
	PolII	11,094,266	9,039,214	81.48	0.72
	INPUT	22,246,057	12,597,695	56.63	1
**E-16-20h**	CBP	15,011,785	4,178,169	27.83	1.07

	K27Ac	10,500,876	8,460,869	80.57	2.17
	K27Me3	10,068,815	7,983,811	79.29	2.04
	K4Me1	18,642,862	7,691,877	41.26	1.97
	K4Me3	9,949,289	7,728,486	77.68	1.98
	K9Ac	9,089,418	7,521,128	82.75	1.92
	K9Me3	10,821,605	8,717,435	80.56	2.23
	PolII	6,534,318	4,398,472	67.31	1.13
	INPUT	8,896,367	3,907,940	43.93	1
**E-20-24h**	CBP	11,293,399	2,354,824	20.85	1.2
	K27Ac	15,088,218	7,290,662	48.32	3.7
	K27Me3	5,236,459	4,292,333	81.97	2.18
	K4Me	15,384,647	8,375,840	54.44	4.26
	K4Me3	10,328,375	6,621,688	64.11	3.36
	K9Ac	6,694,031	4,739,350	70.8	2.41
	K9Me3	5,048,272	3,007,623	59.58	1.53
	PolII	6,471,911	3,282,860	50.72	1.67
	INPUT	4,842,413	1,968,057	40.64	1
**E-4-8h**	CBP-seq1	3,990,294	2,883,861	72.27	0.32
	CBP-seq2	9,138,780	6,610,999	72.34	0.74
	INPUT	13,200,940	8,903,620	67.45	1
**AdultMale**	CBP-seq1	14,018,596	10,180,396	72.62	0.76
	CBP-seq2	2,368,959	1,885,739	79.6	0.14
	INPUT	28,392,197	13,453,639	47.38	1
**AdultFemale**	CBP-seq1	15,467,826	11,738,932	75.89	4.01
	CBP-seq2	1,912,977	1,534,125	80.2	0.52
	INPUT	4,500,253	2,925,836	65.01	1
**CTCF, S2 cell**	CTCF 200	7,904,780	7,318,867	92.59	0.97
	INPUT	8,007,425	7,552,105	94.31	1
	12.6M)	8,848,308	8,078,587	91.3	1.29
	INPUT	6,682,773	6,246,278	93.47	1

Table S3. Summary of the INPUT-chip and INPUT-seq

Platform	Symbol	Biological sample
**INPUT-chip**	**E0a**	E-0-4h, H3K4Me3
	**E0b**	E-0-4h, H3K4Me1
	**E12a**	E-12-16h, H3K4Me3
	**E12b**	E-12-16h, H3K4Me1
	**E16a**	E-16-20h, H3K4Me3
	**E16b**	E-16-20h, H3K4Me1
	**E20a**	E-20-24h, H3K4Me3
	**E20b**	E-20-24h, H3K4Me1
**INPUT-seq**	**E0**	E-0-4h
	**E4**	E-4-8h
	**E12**	E-12-16h
	**E16**	E-16-20
	**E20**	E-20-24h
	**AM**	AdultMale
	**AF**	AdultFemale
	**C200**	CTCF 200
	**C500**	CTCF 500

Table S4a. Skewness of the ChIP-chip density profiles

IP	Stage	Skewness, Seq	Skewness, chip1	Skewness, chip2	Skewness, chip3
CBP	E-0-4h	-0.03	0.00	-0.08	0.09
CBP	E-16-20h	0.92	0.02	0.00	0.04
CBP	E-20-24h	1.29	0.08	0.15	0.17
H3K4Me1	E-0-4h	0.12	0.18	0.16	0.20
H3K4Me1	E-12-16h	0.59	0.40	0.38	0.47
H3K4Me1	E-16-20h	0.33	0.04	0.04	0.06
H3K4Me1	E-20-24h	0.08	0.01	0.00	0.01
H3K4Me3	E-0-4h	0.18	0.17	0.16	0.15
H3K4Me3	E-12-16h	0.28	0.45	0.14	0.28
H3K4Me3	E-16-20h	0.54	0.16	0.08	0.06
H3K4Me3	E-20-24h	0.25	0.16	0.09	0.22
H3K27Ac	E-0-4h	0.07	0.21	0.21	0.01
H3K27Ac	E-12-16h	0.27	0.35	0.39	0.38
H3K27Ac	E-16-20h	0.22	0.09	0.09	0.09
H3K27Ac	E-20-24h	0.13	0.10	0.07	0.06
H3K27Me3	E-0-4h	0.74	0.27	0.24	0.26
H3K27Me3	E-12-16h	1.86	1.23	1.12	1.16
H3K27Me3	E-16-20h	0.76	0.46	0.31	0.56
H3K27Me3	E-20-24h	0.49	0.22	0.28	0.24
H3K9Ac	E-0-4h	0.21	-0.10	-0.10	-0.16
H3K9Ac	E-12-16h	0.35	0.17	0.16	0.14
H3K9Ac	E-16-20h	0.05	-0.09	-0.05	-0.05
H3K9Ac	E-20-24h	0.20	-0.09	-0.08	-0.09
H3K9Me3	E-0-4h	0.32	0.26	0.24	0.23
H3K9Me3	E-12-16h	0.75	0.76	0.86	0.42
H3K9Me3	E-16-20h	0.60	0.52	0.48	0.38
H3K9Me3	E-20-24h	0.28	0.24	0.23	0.24
PolII	E-0-4h	0.12	-0.07	-0.02	0.01
PolII	E-12-16h	0.23	0.12	0.08	0.10
PolII	E-16-20h	0.24	-0.03	0.02	0.11
PolII	E-20-24h	-0.11	-0.68	-0.03	-0.14

Table S4b. Skewness of the ChIP-seq profiles

Seq. vs. Seq	skewSeq1	skewSeq2
E-4-8h, CBP,	0.22	0.23
AdultMale, CBP,	1.50	1.18
AdultFemale, CBP	0.75	0.64
S2, CTCF	0.23	0.53

Table S5a. Correlation coefficient between every pair of ChIP-chip replicate profiles

IP	Stage	Correlation, seq vs. chip	Correlation, chip1 vs. 2	Correlation, chip1 vs. 3	Correlation, chip2 vs.3
CBP	E-0-4h	0.24	0.33	0.88	0.33
CBP	E-16-20h	0.62	0.86	0.86	0.87
CBP	E-20-24h	0.47	0.78	0.78	0.82
H3K4Me1	E-0-4h	0.40	0.94	0.97	0.94
H3K4Me1	E-12-16h	0.62	0.95	0.96	0.96
H3K4Me1	E-16-20h	0.50	0.88	0.88	0.91
H3K4Me1	E-20-24h	0.42	0.85	0.88	0.83
H3K4Me3	E-0-4h	0.49	0.90	0.89	0.89
H3K4Me3	E-12-16h	0.69	0.84	0.89	0.80
H3K4Me3	E-16-20h	0.29	0.92	0.92	0.99
H3K4Me3	E-20-24h	0.36	0.71	0.84	0.85
H3K27Ac	E-0-4h	0.48	0.97	0.85	0.85
H3K27Ac	E-12-16h	0.26	0.88	0.87	0.88
H3K27Ac	E-16-20h	0.22	0.75	0.73	0.73
H3K27Ac	E-20-24h	0.36	0.89	0.89	0.85
H3K27Me3	E-0-4h	0.56	0.77	0.79	0.76
H3K27Me3	E-12-16h	0.83	0.97	0.98	0.96
H3K27Me3	E-16-20h	0.55	0.94	0.97	0.94
H3K27Me3	E-20-24h	0.32	0.99	0.99	0.99
H3K9Ac	E-0-4h	0.48	0.69	0.67	0.63
H3K9Ac	E-12-16h	0.41	0.90	0.79	0.77
H3K9Ac	E-16-20h	0.27	0.87	0.85	0.89
H3K9Ac	E-20-24h	-0.19	0.81	0.87	0.67
H3K9Me3	E-0-4h	0.45	0.61	0.61	0.61
H3K9Me3	E-12-16h	0.63	0.90	0.81	0.83
H3K9Me3	E-16-20h	0.51	0.91	0.84	0.83
H3K9Me3	E-20-24h	0.36	0.83	0.81	0.83
PolII	E-0-4h	0.17	0.72	0.73	0.76
PolII	E-12-16h	0.01	0.43	0.56	0.97
PolII	E-16-20h	0.23	0.46	0.52	0.76
PolII	E-20-24h	0.14	0.30	0.84	0.44

Table S5b. Correlation coefficient between every pair of ChIP-seq replicate profiles

Seq. vs. Seq	Correlation
E-4-8h, CBP	0.72
AdultMale, CBP	0.93
AdultFemale, CBP	0.93
S2, CTCF	0.71

Table S6. Probe level (Pearson) correlation (mean and standard deviation) between pairs of biological replicates for ChIP-chip

	E-0-4h		E12-16h		E-16-20h		E-20-24h	
	Mean	SD	Mean	SD	Mean	SD	Mean	SD
**CBP**	0.47	0.26	---	---	0.76	0.03	0.68	0.03
**H3K27Ac**	0.85	0.08	0.86	0.01	0.69	0.02	0.84	0.03
**H3K27Me3**	0.63	0.02	0.92	0.01	0.91	0.02	0.98	0.00
**H3K4Me1**	0.92	0.03	0.92	0.02	0.84	0.03	0.79	0.03
**H3K4Me3**	0.84	0.01	0.88	0.03	0.93	0.04	0.78	0.07
**H3K9Ac**	0.57	0.03	0.79	0.07	0.77	0.02	0.75	0.09
**H3K9Me3**	0.46	0.00	0.75	0.05	0.77	0.04	0.72	0.01
**PolII**	0.22	0.38	0.68	0.24	0.56	0.15	0.54	0.23

Table S7. Functions and parameters used for ChIP-seq preprocessing using SPP

Step	Function called	Parameters
Construction of cross-correlation profile	get.binding.characteristics	Srange=c(30,300), bin=5
Selection of informative tags	select.informative.tags	Default parameters
Removal of local tag anomaly	remove.local.tag.anomalies	Default parameters
Peak calling	find.binding.positions *followed by* Add.broad.peak.regions	Default parameters

Table S8. Peak caller parameters

Seq/chip	Method	Parameters
ChIP-seq	SPP	See Supplemental Table 7
	MACS	Band width=300, model fold=32, pvalue cutoff=1.00e-02
ChIP-chip	MA2C	Robust normalization, C=2, Pvalue cut off = 1e-2, bandwidth=500, min_probes=5, max_gap=250
	Splitters	Perform normalization between replicate: None Signal cutoff >= 5%le Gap (Maxgap) <= 100 bp Clustering (Minrun) >= 5 probes

Table S9. Information about the Agilent custom tiling microarray used in this study. The ChIP-chip experiments presented in this study was performed using Agilent's custom tiling microarrays. Three microarrays are required to cover one *D. melanogaster* genome. Each array contains about 244,000 probes and each probe is 45bp-60bp long. The information about these microarrays can be found at the NCBI Gene Expression Omnibus (GEO) using the following accession numbers and URLs.

Microarray	GEO platform accession number	URL
Array 1	GPL6949	http://www.ncbi.nlm.nih.gov/geo/query/acc.cgi?acc=GPL6949
Array 2	GPL6950	http://www.ncbi.nlm.nih.gov/geo/query/acc.cgi?acc=GPL6950
Array 3	GPL6951	http://www.ncbi.nlm.nih.gov/geo/query/acc.cgi?acc=GPL6949
