# Supplementary material for: ChIP-chip versus ChIP-seq: Lessons for experimental design and data analysis
Source: BMC Genomics. 2011 Feb 28;12:134. doi: 10.1186/1471-2164-12-134 (PMC3053263; doi:10.1186/1471-2164-12-134)
Supplement: Additional file 2 — Supplemental figures. This file contains supplementary figures. [file 1471-2164-12-134-S2.PDF]

Supplemental Figures for "ChIP-chip versus ChIP-seq: Lessons for experimental design and data analysis"

Joshua W.K. Ho, Eric Bishop, Peter V. Kharchenko, Nicolas Nègre, Kevin P. White, Peter J. Park

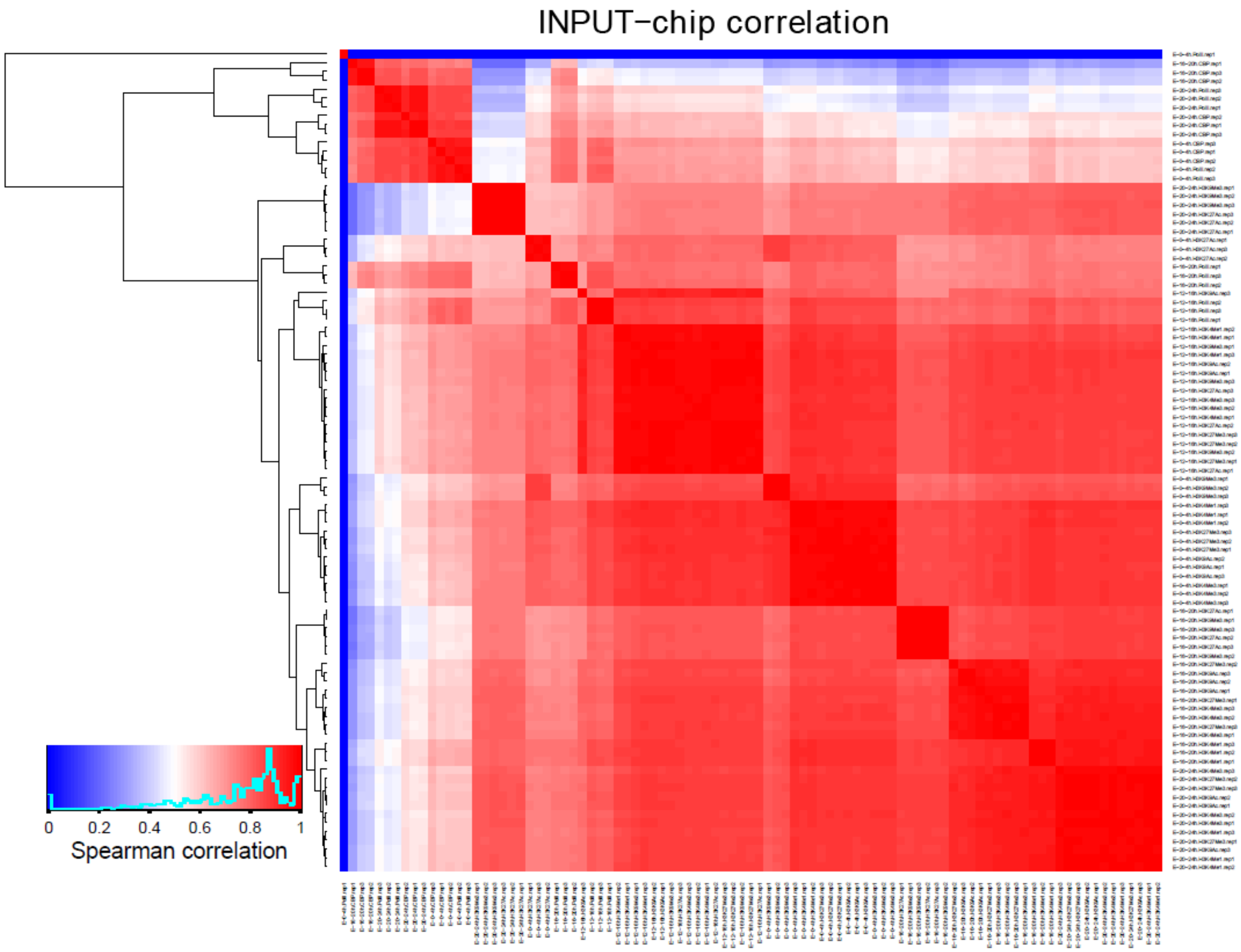

Figure S1. A heat map summary representation of the Spearman correlations between all pairs of INPUT-chip profiles at the probe level. Majority of the profiles clustered together tightly. The eight INPUT-chip profiles analyzed in this study were selected to be representatives of the full set of INPUT-chip profiles.

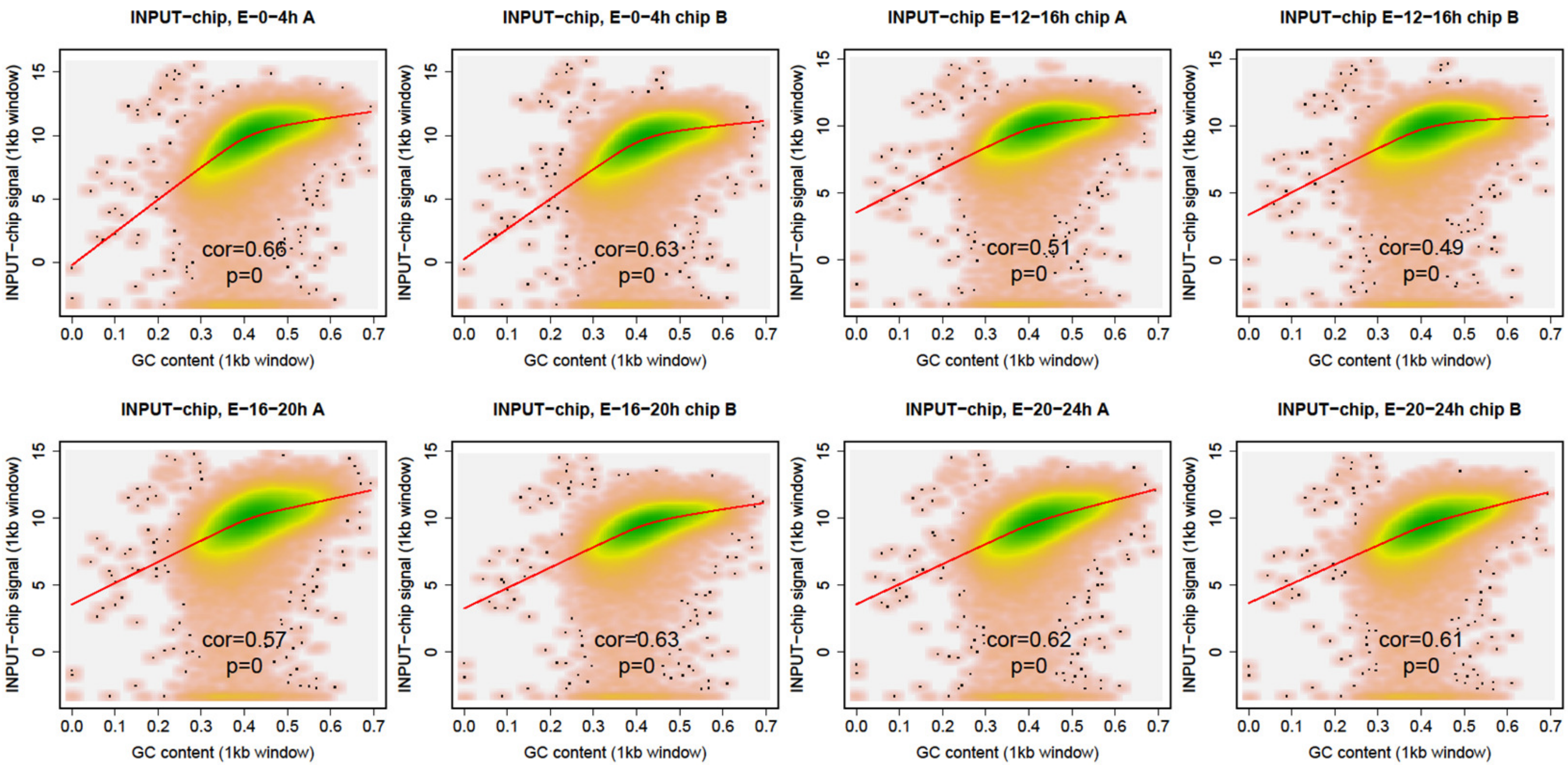

Figure S2a. Genome-wide (Spearman) correlation between INPUT-chip and GC content. A LOESS fitted line is superimposed on every scatter plot.

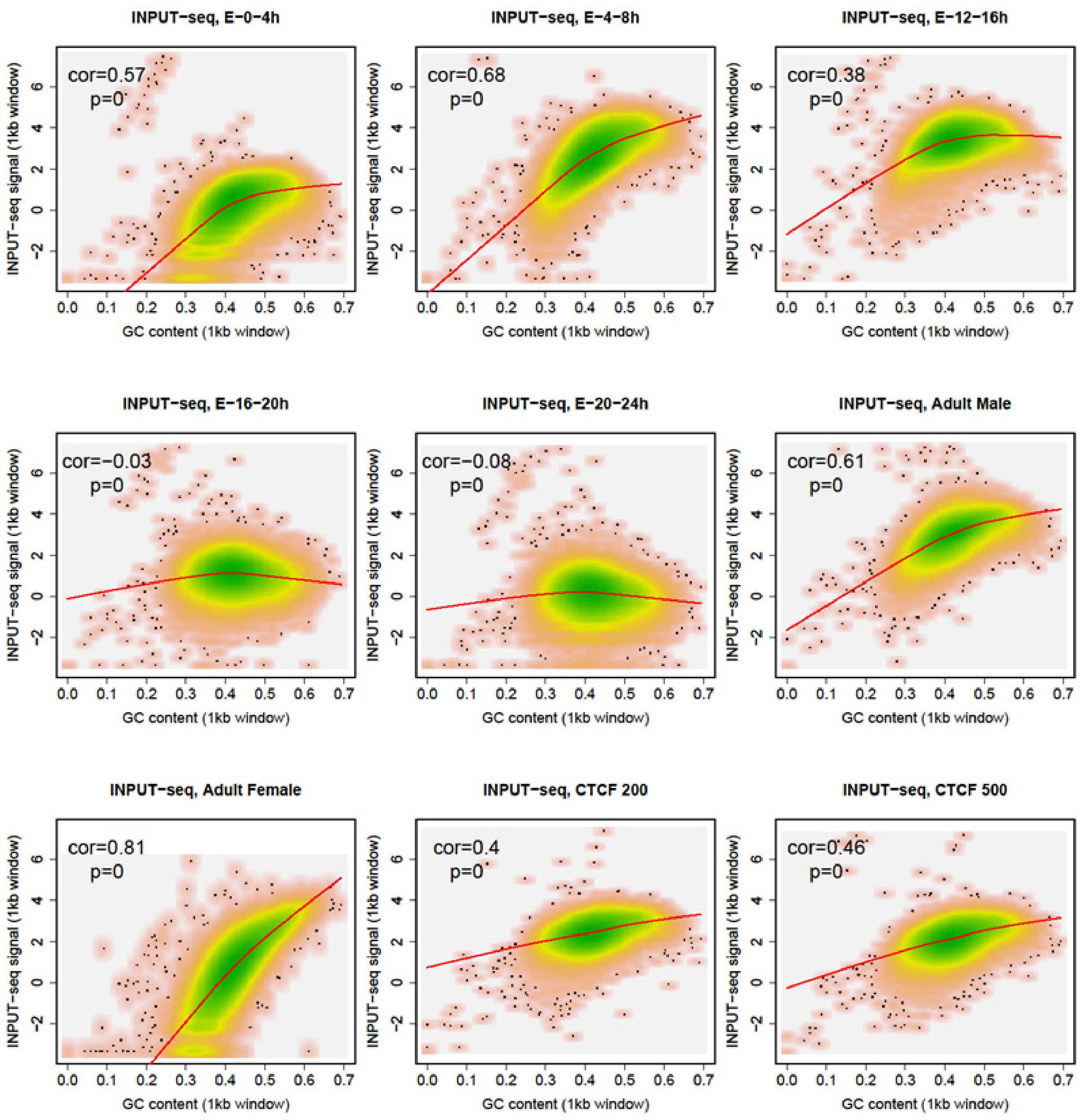

Figure S2b. Genome-wide (Spearman) correlation between INPUT-seq and GC content. A LOESS fitted line is superimposed on every scatter plot.

Label	Subsample proportion	number of million reads
**AM**	100	13.45
**AM90**	90	12.11
**AM80**	80	10.76
**AM70**	70	9.42
**AM60**	60	8.07
**AM50**	50	6.73
**AM40**	40	5.38
**AM30**	30	4.04
**AM20**	20	2.69
**AM10**	10	1.35
**AM5**	5	0.67
**AM1**	1	0.13

Figure S3. To investigate the relationship between sequencing depth and correlation of INPUT-seq profiles, we generated 11 addition profiles from one of the INPUT-seq sample (AM) by subsampling the reads at different proportion (90%,80%,...,10%,5%,1%).

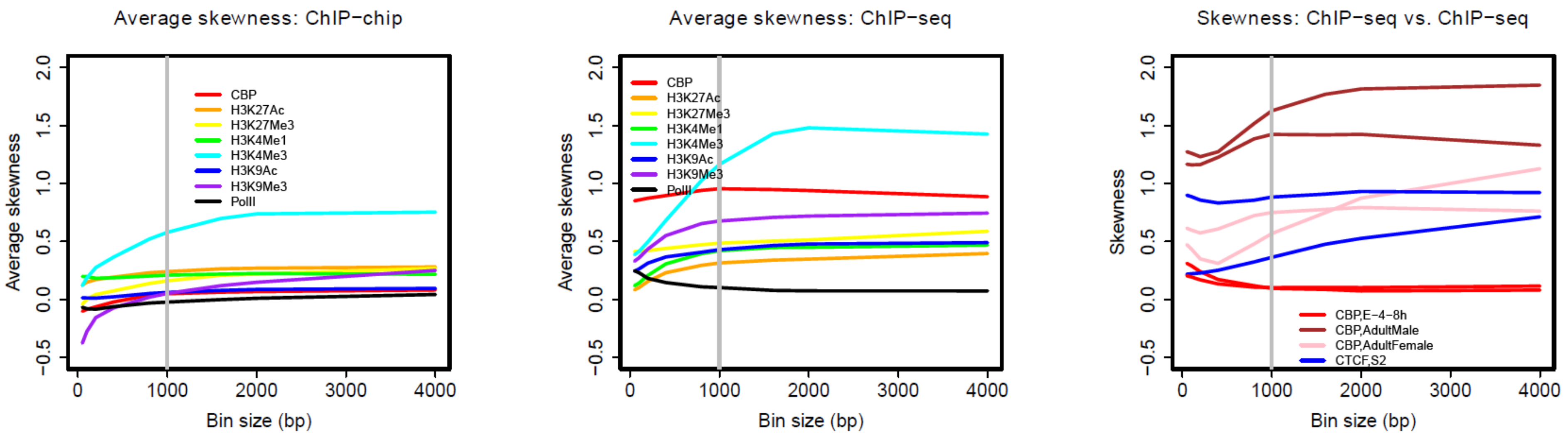

Figure S4. The relative signal distribution skewness is largely consistent with different bin size.

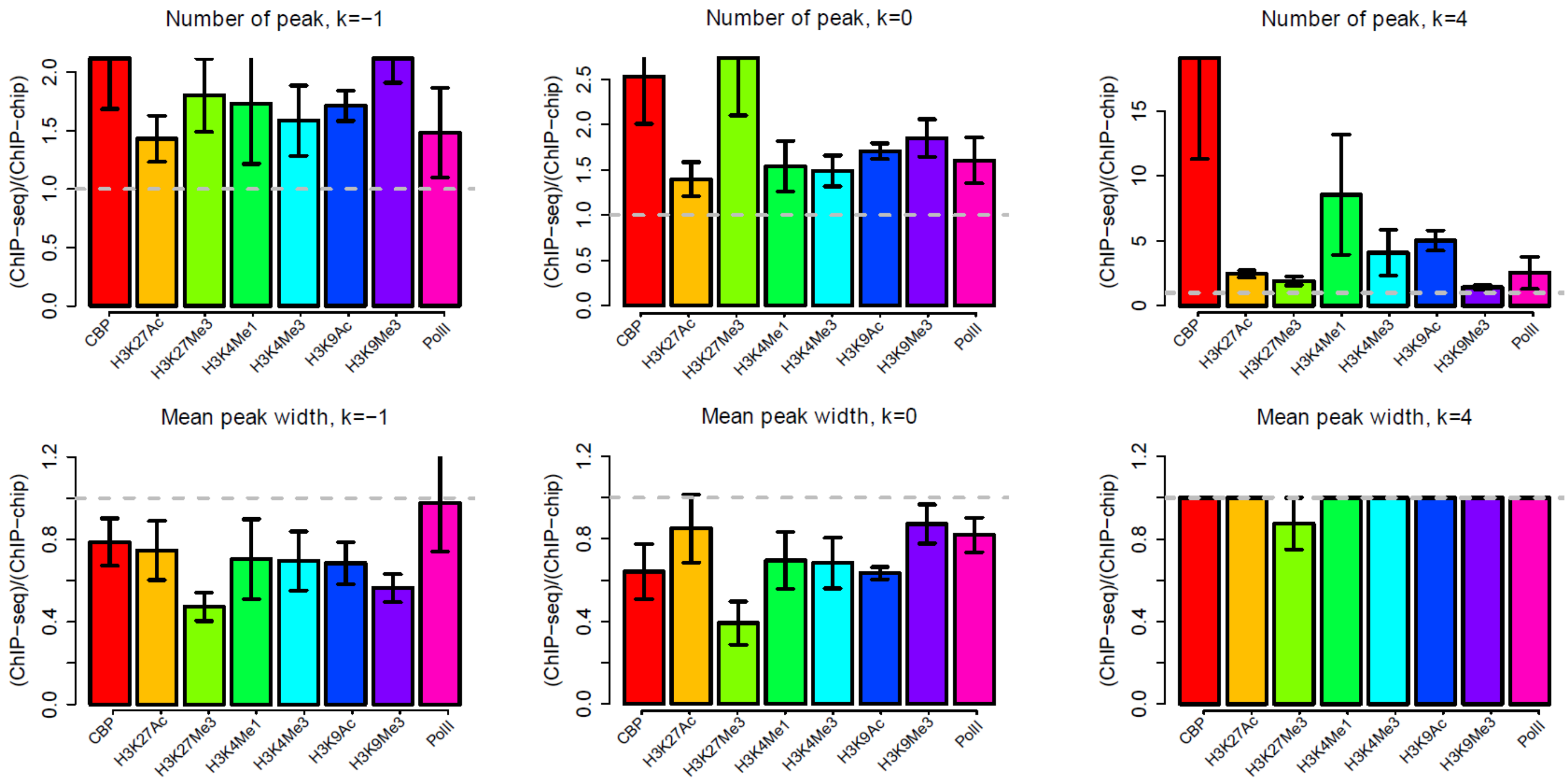

Figure S5. Comparative analysis of the number of peaks and mean peak width generated from ChIP-seq and ChIP-chip profiles. The conclusion that ChIP-seq generates more, and narrower peaks are supported with a range of analysis parameters (*k*=-1, 0, and 4).

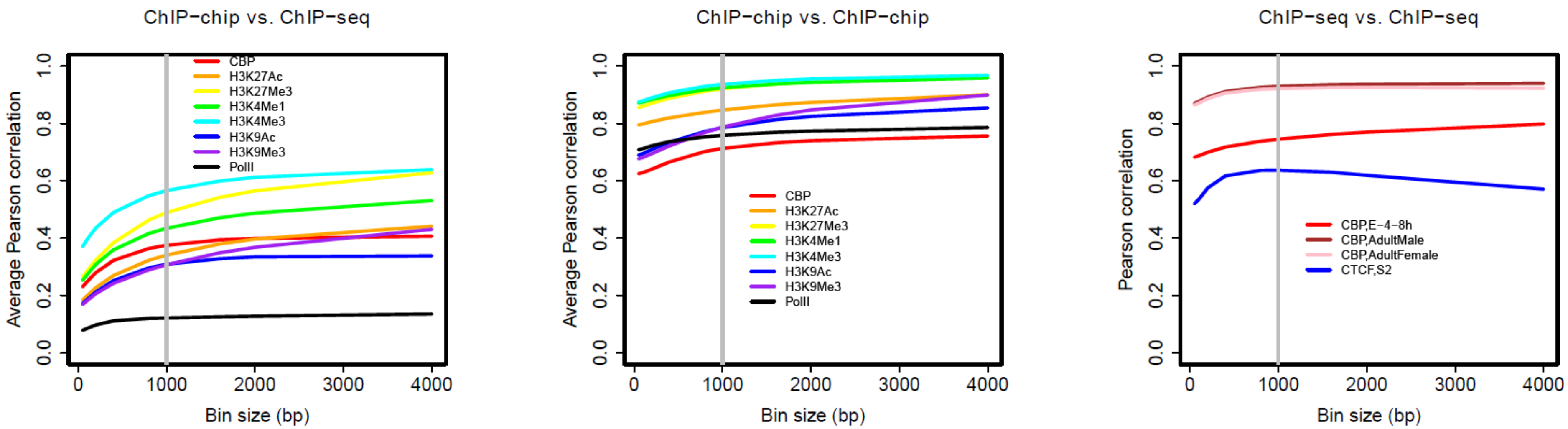

Figure S6. The relative pairwise genome-wide correlations between profiles are consistent with bin size.

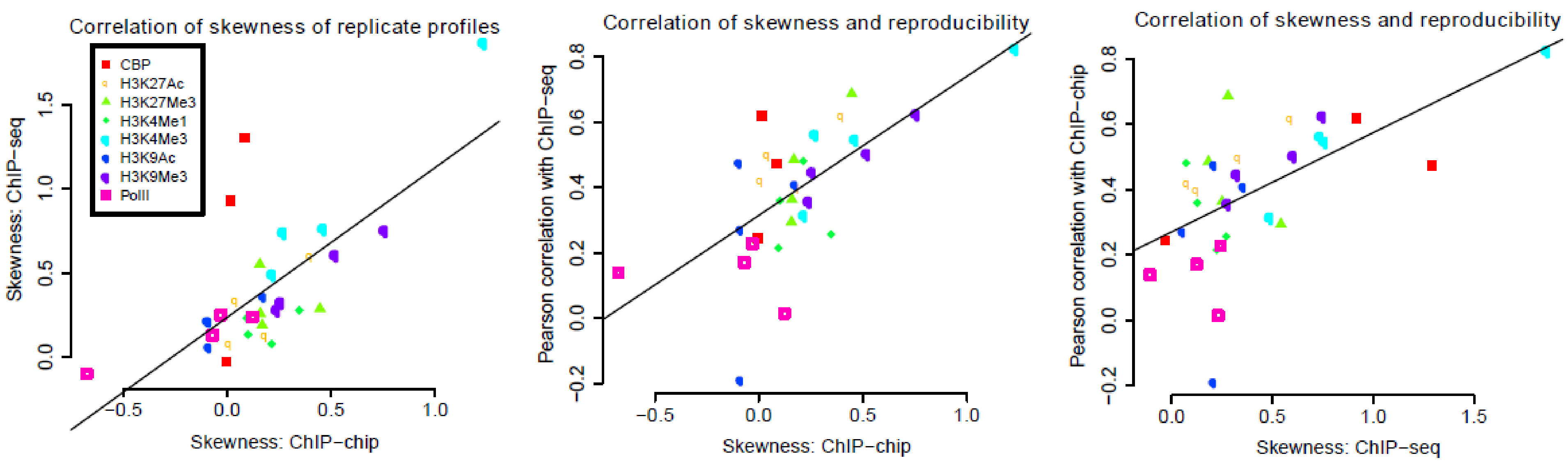

Figure S7. Signal distribution skewness is positively correlated among ChIP-chip/seq replicates, and with genome-wide reproducibility (as measured by Pearson correlation coefficient with its replicate profile). This result suggests that a more sensitive antibody generally produce consistent profiles between the two technologies.

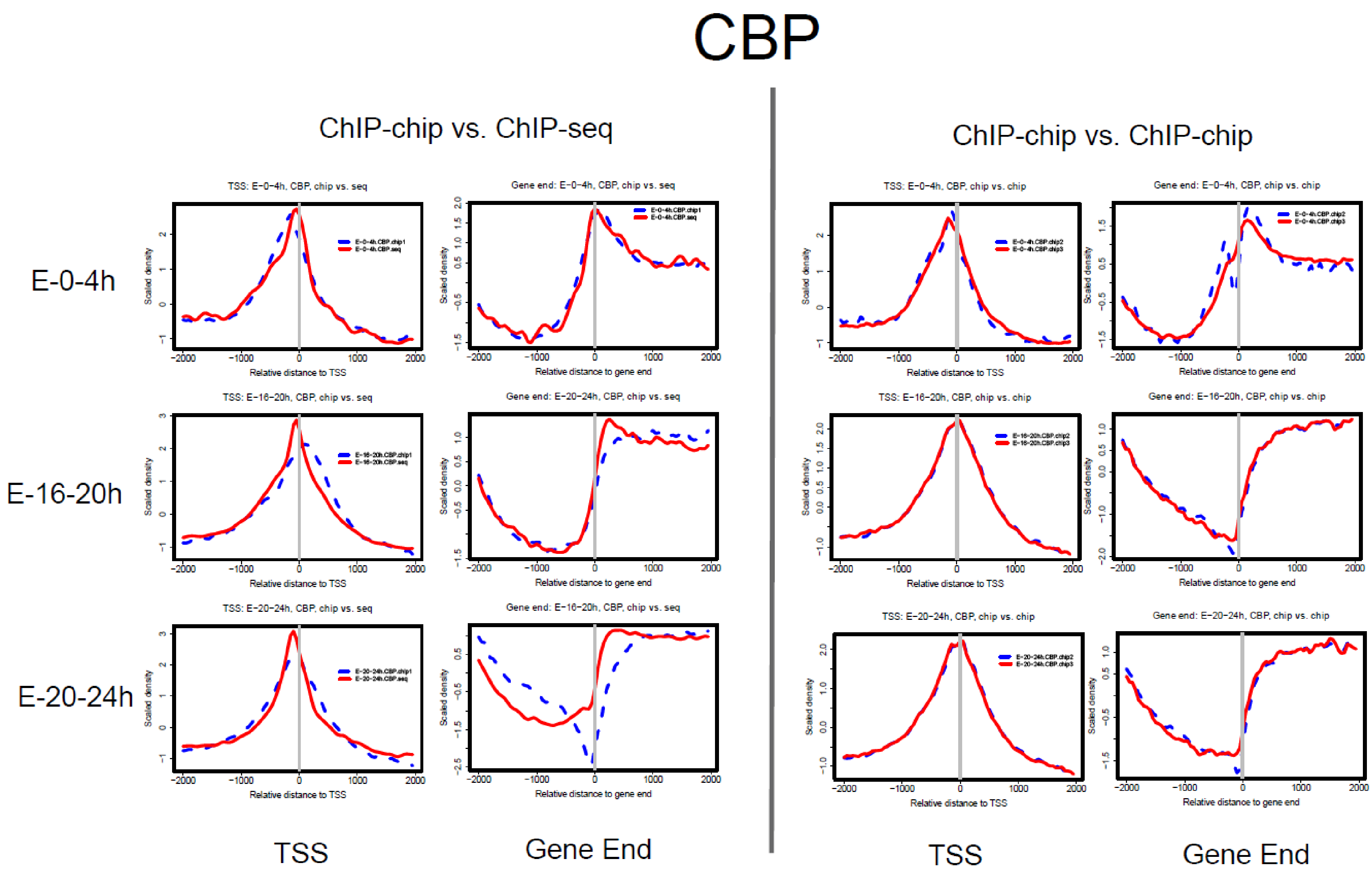

Figure S8a. Average TSS and TES (gene end) profiles of CBP.

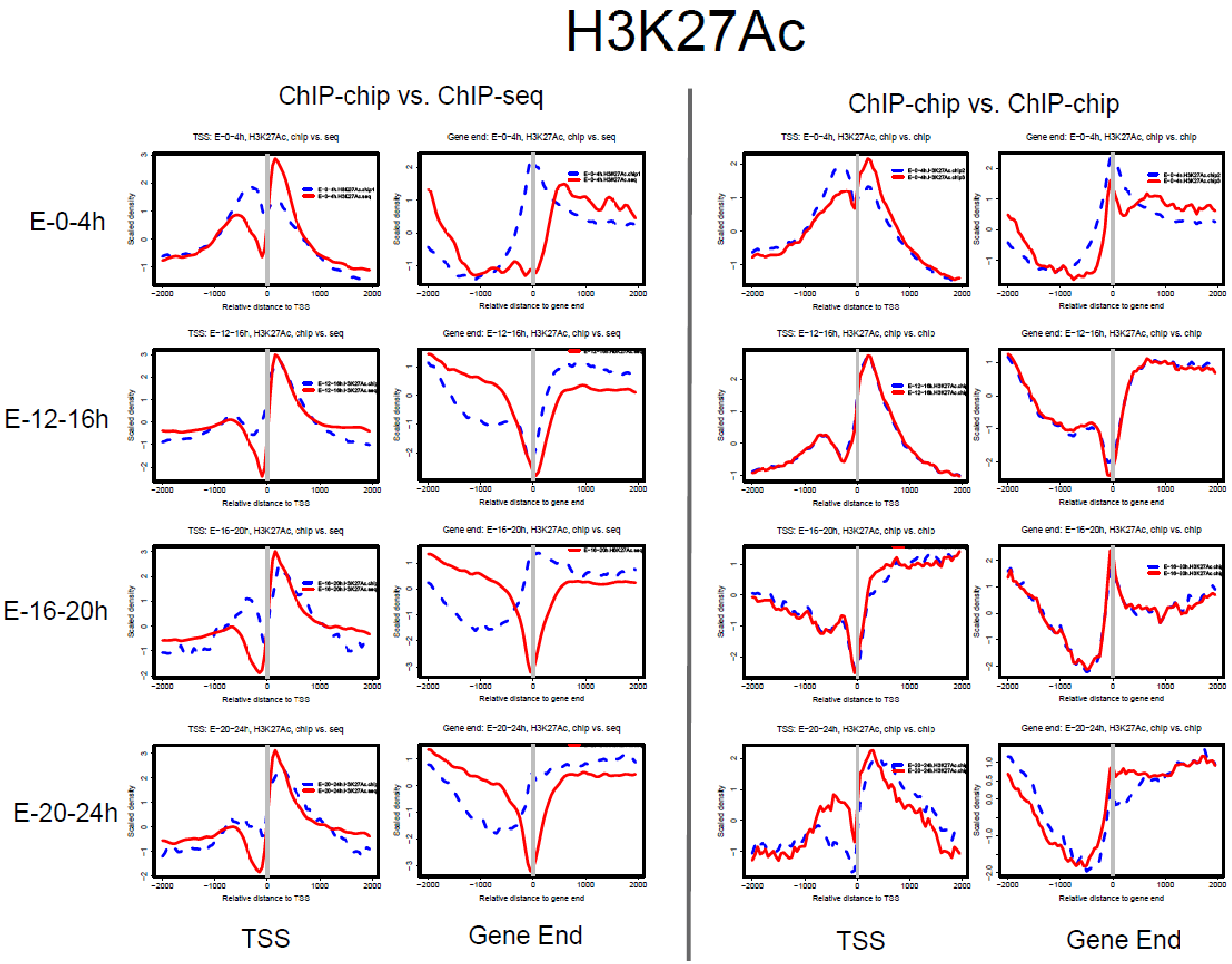

Figure S8b. Average TSS and TES (gene end) profiles of H3K27Ac.

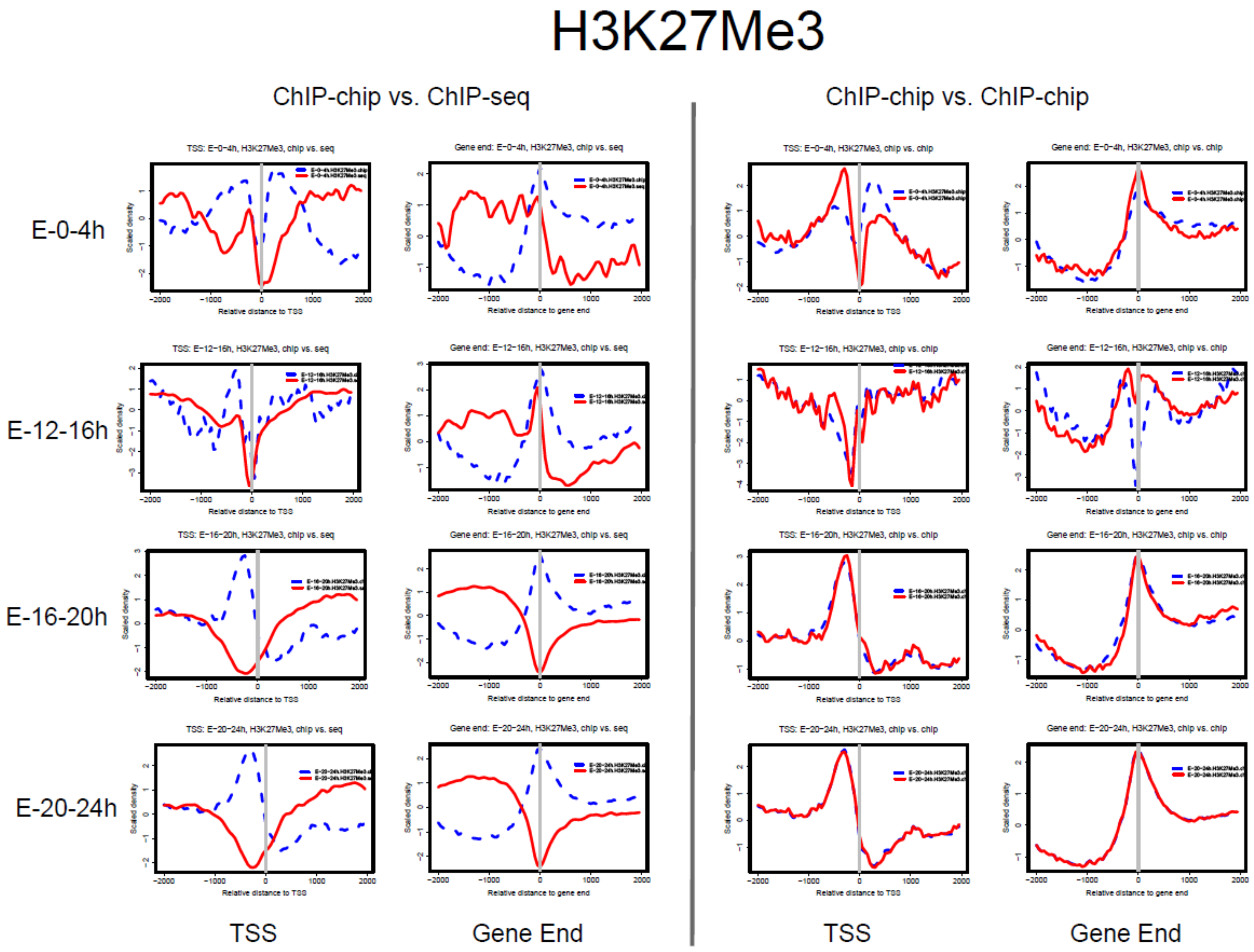

Figure S8c. Average TSS and TES (gene end) profiles of H3K27Me3.

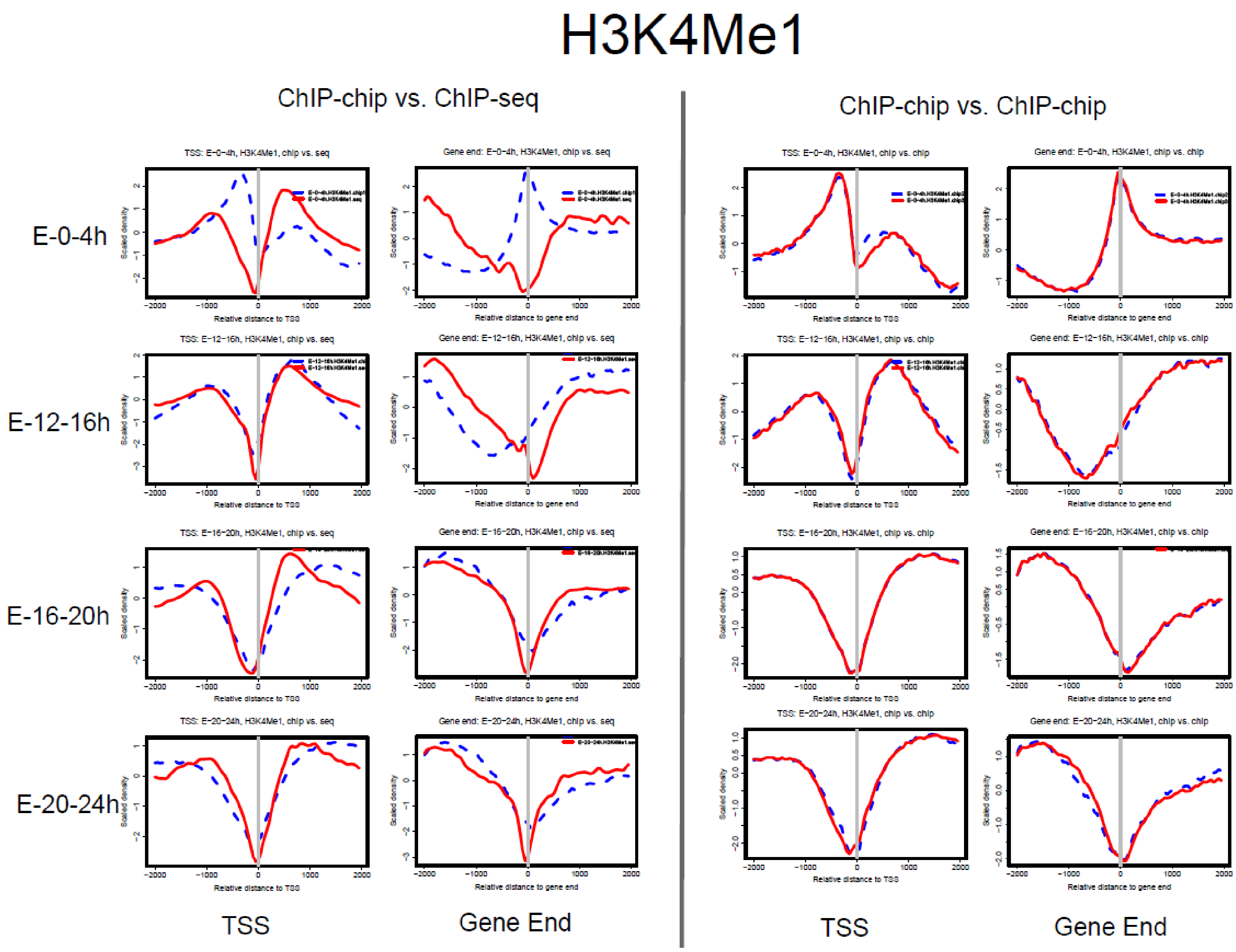

Figure S8d. Average TSS and TES (gene end) profiles of H3K4Me1.

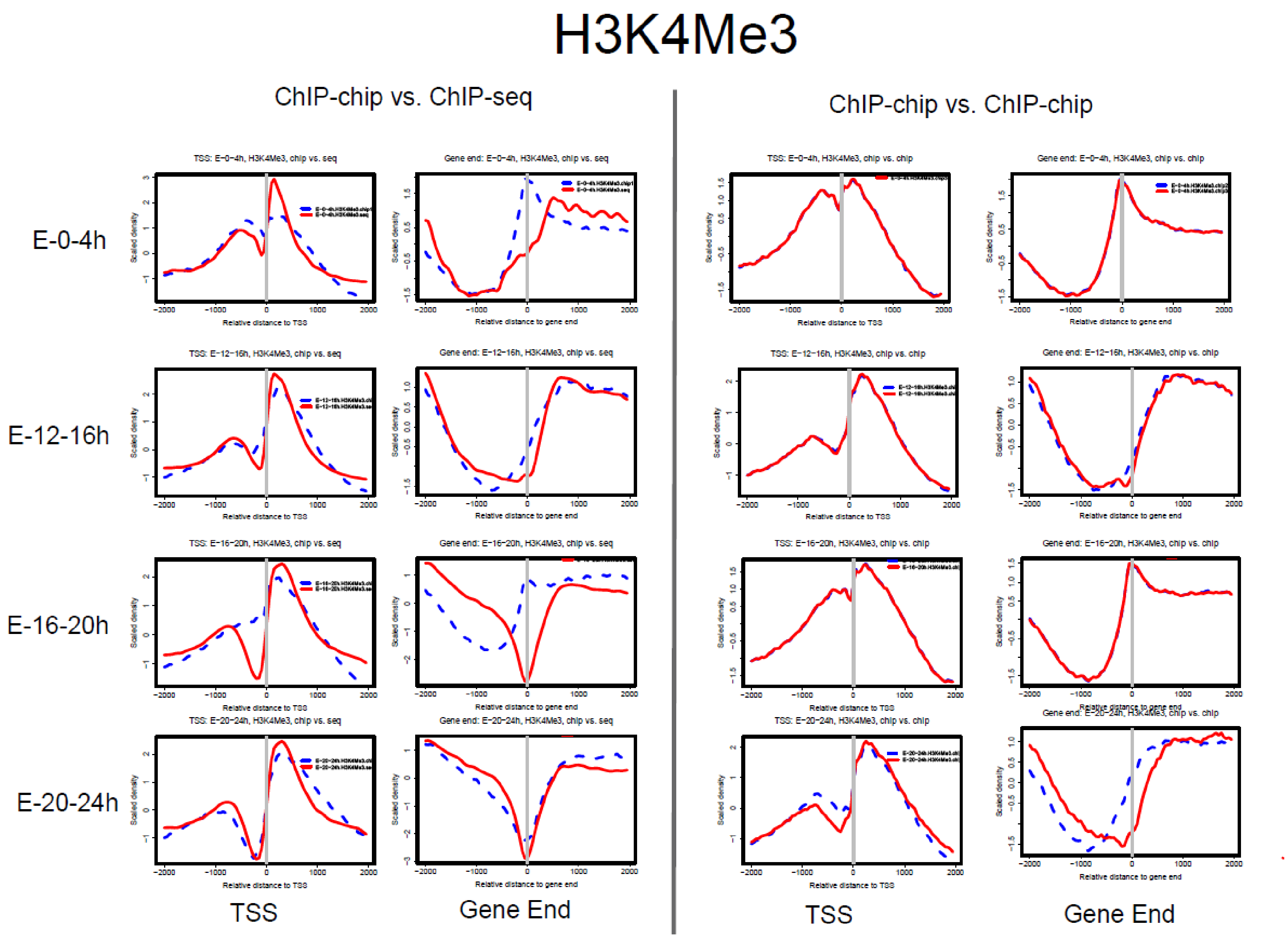

Figure S8e. Average TSS and TES (gene end) profiles of H3K4Me3.

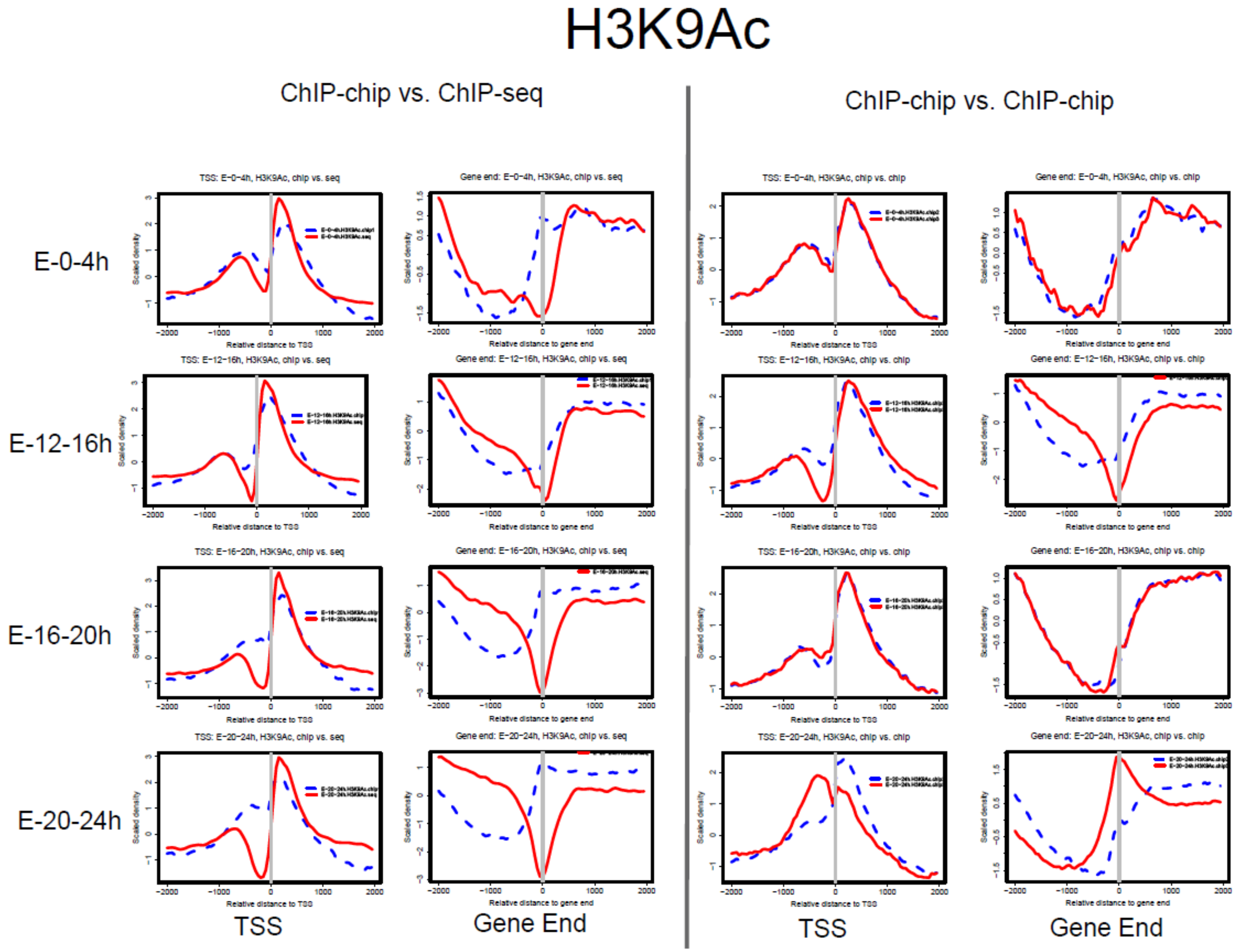

Figure S8f. Average TSS and TES (gene end) profiles of H3K9Ac.

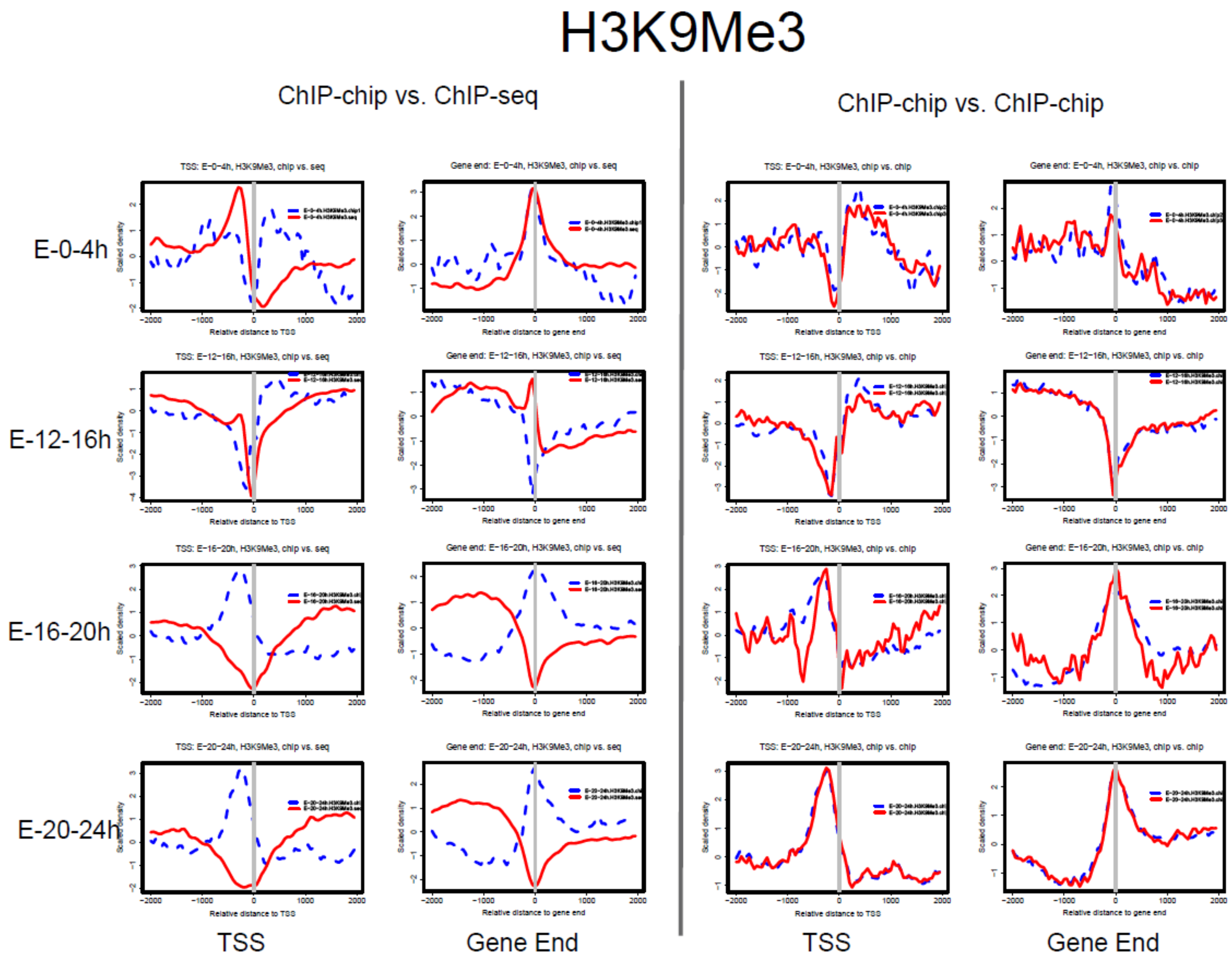

Figure S8g. Average TSS and TES (gene end) profiles of H3K9Me3.

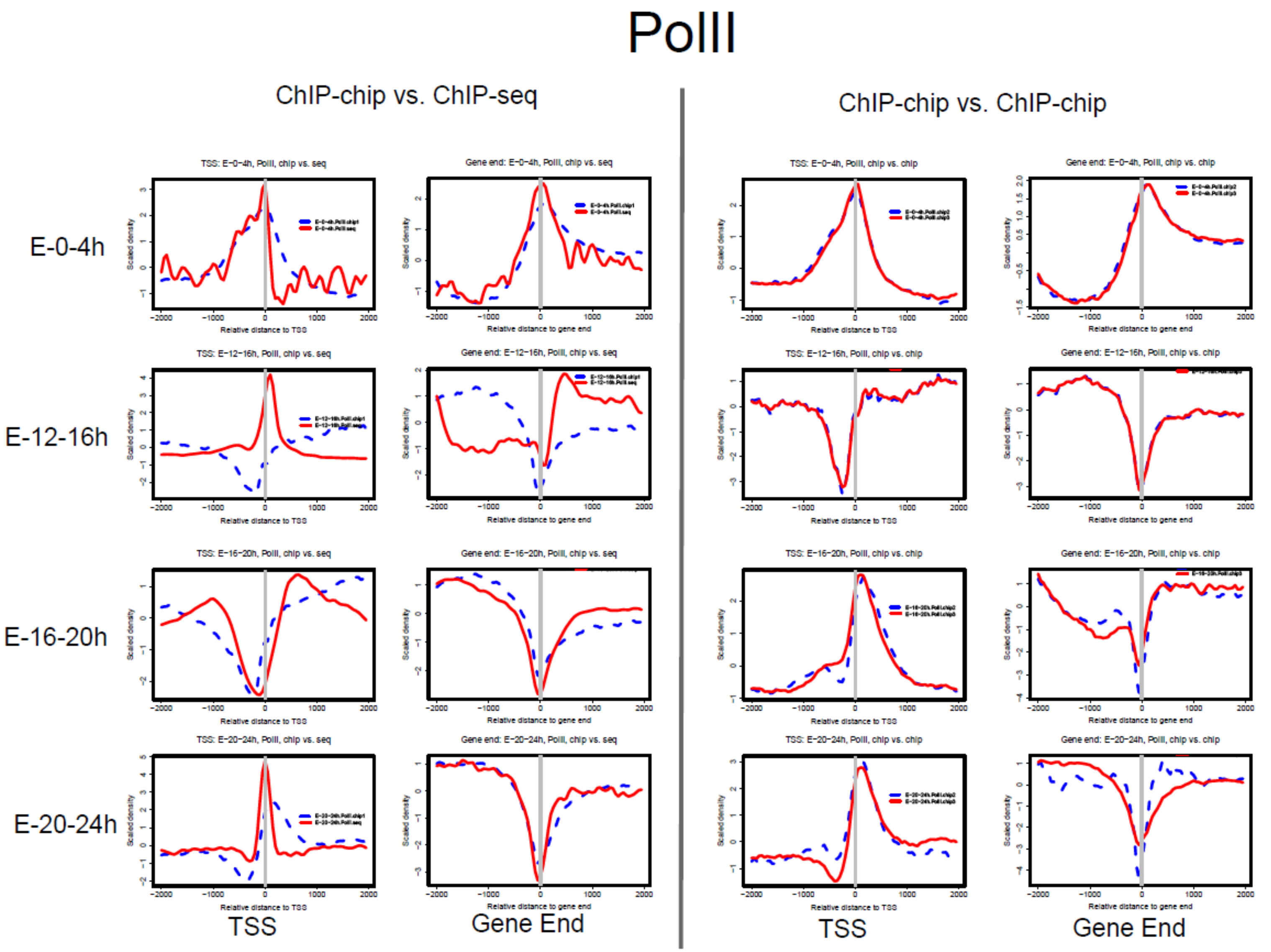

Figure S8h. Average TSS and TES (gene end) profiles of PolII.

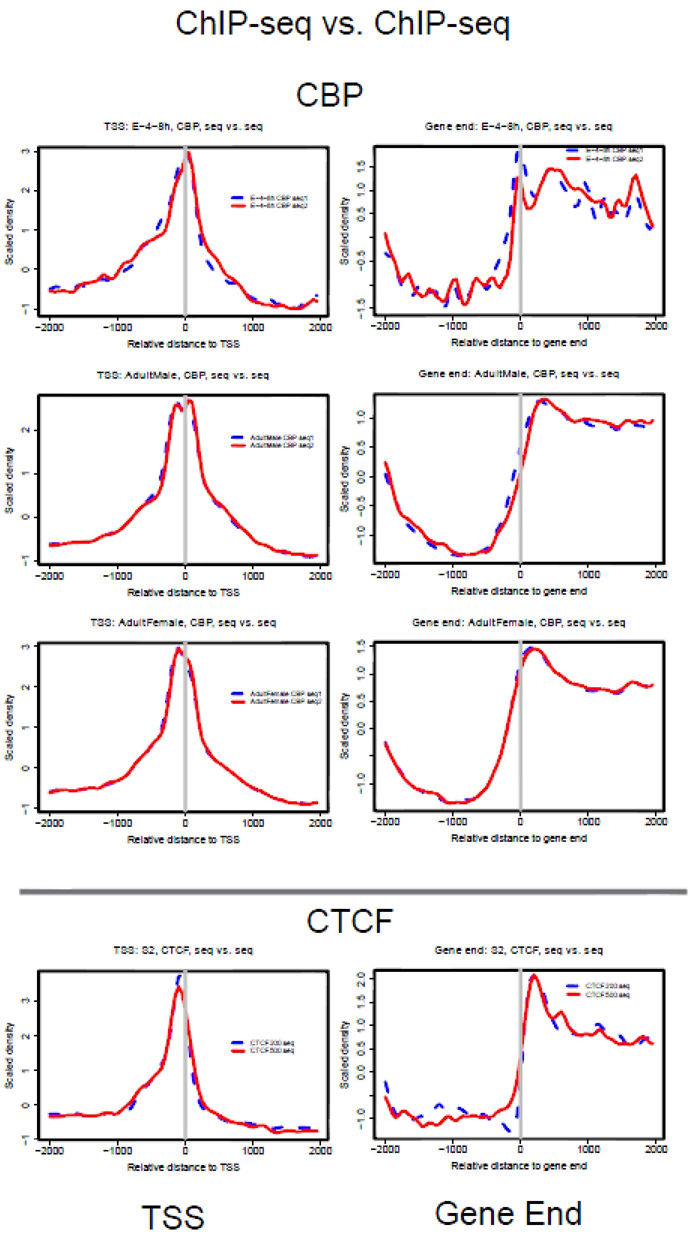

Figure S8i. Average TSS and TES (gene end) profiles of CBP and CTCF using the four pairs of ChIP-seq/ChIP-seq replicates data.

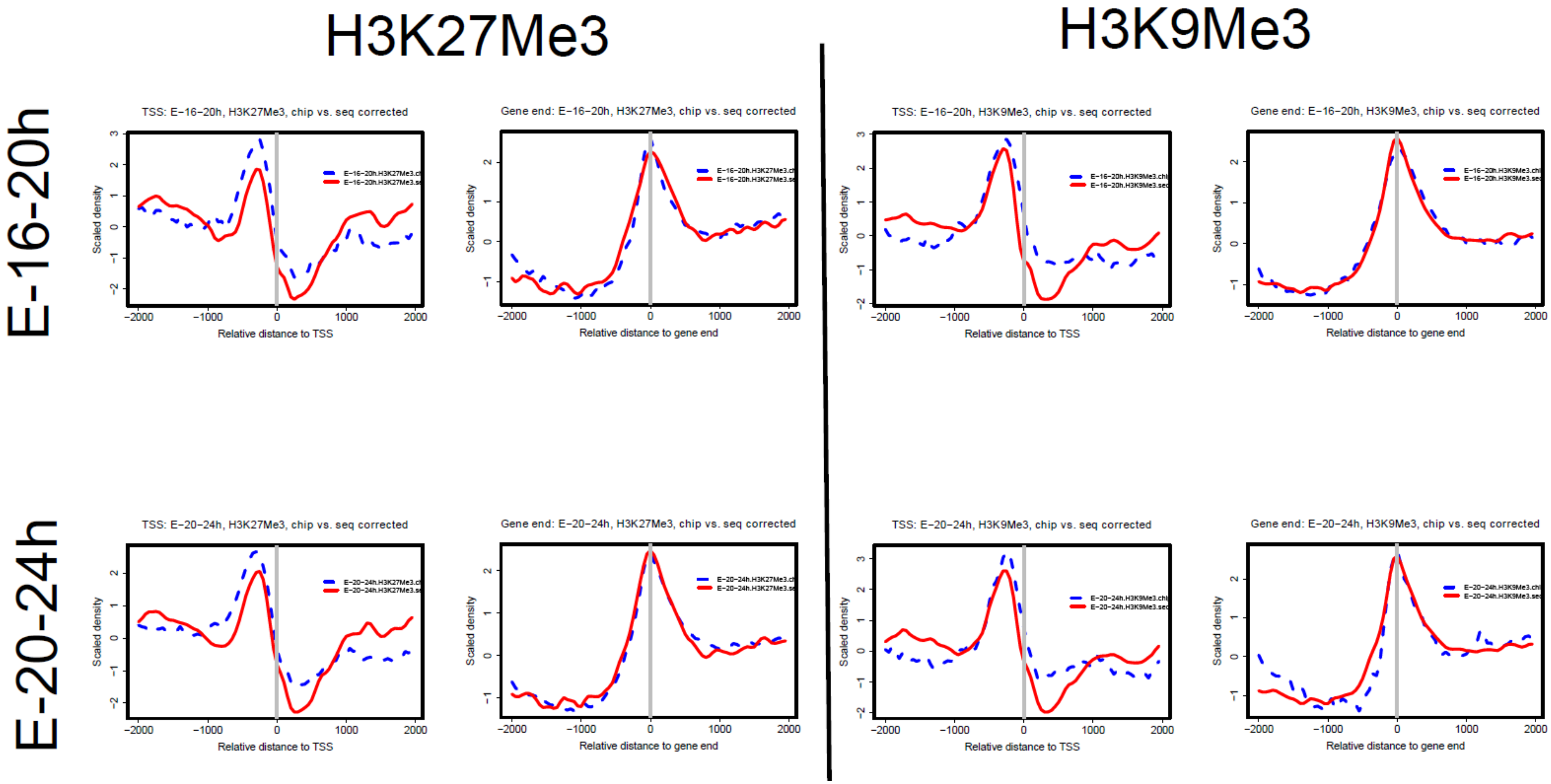

Figure S9. Average TSS and TES (gene end) profiles of H3K27Me3 and H3K9Me3 at E-16-20h and E-20-24h after re-normalization with INPUT-seq AF. The average profiles constructed from the re-normalized ChIP-seq data are clearly more consistent with the profiles generated from ChIP-chip data.

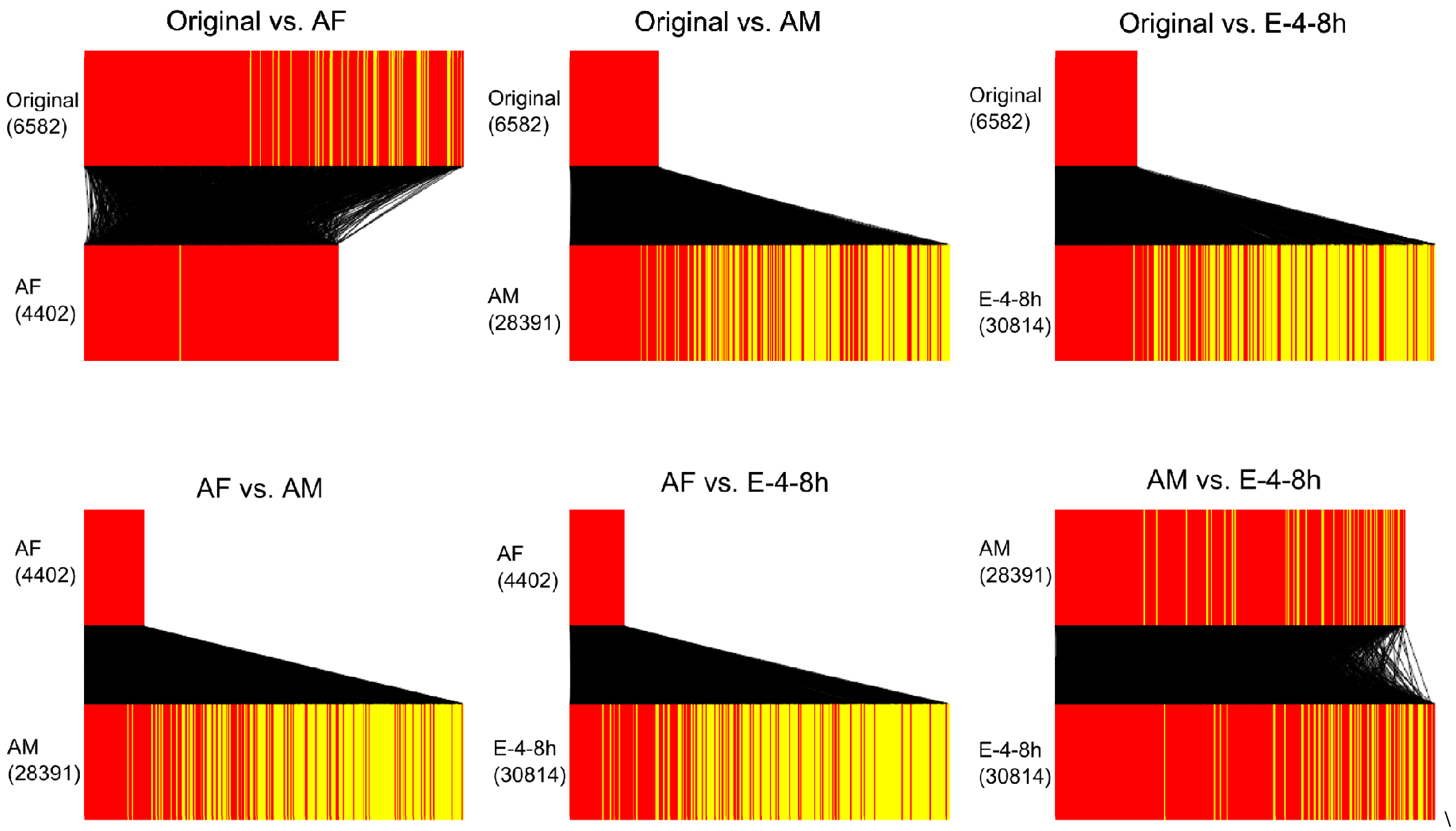

Figure S10. Comparison of the statistically significant peaks (FDR<0.05) identified in the E-20-24h H3K27Me3 ChIP-seq profile using four different input DNA profiles as background (Original = E-20-24h, AF=AdultFemale, AM=AdultMale, E-4-8h). In each box, the red bar represents a common peaks in two peak set, while a yellow bar represents a peak that is only present in one peak set. The peaks (red or yellow bars) in each peak set (the box) is sorted by FDR in ascending order such that the most significant peak is drawn on the left, and so on. In general, the peaks with lower FDR is more likely to overlap with other peak sets.

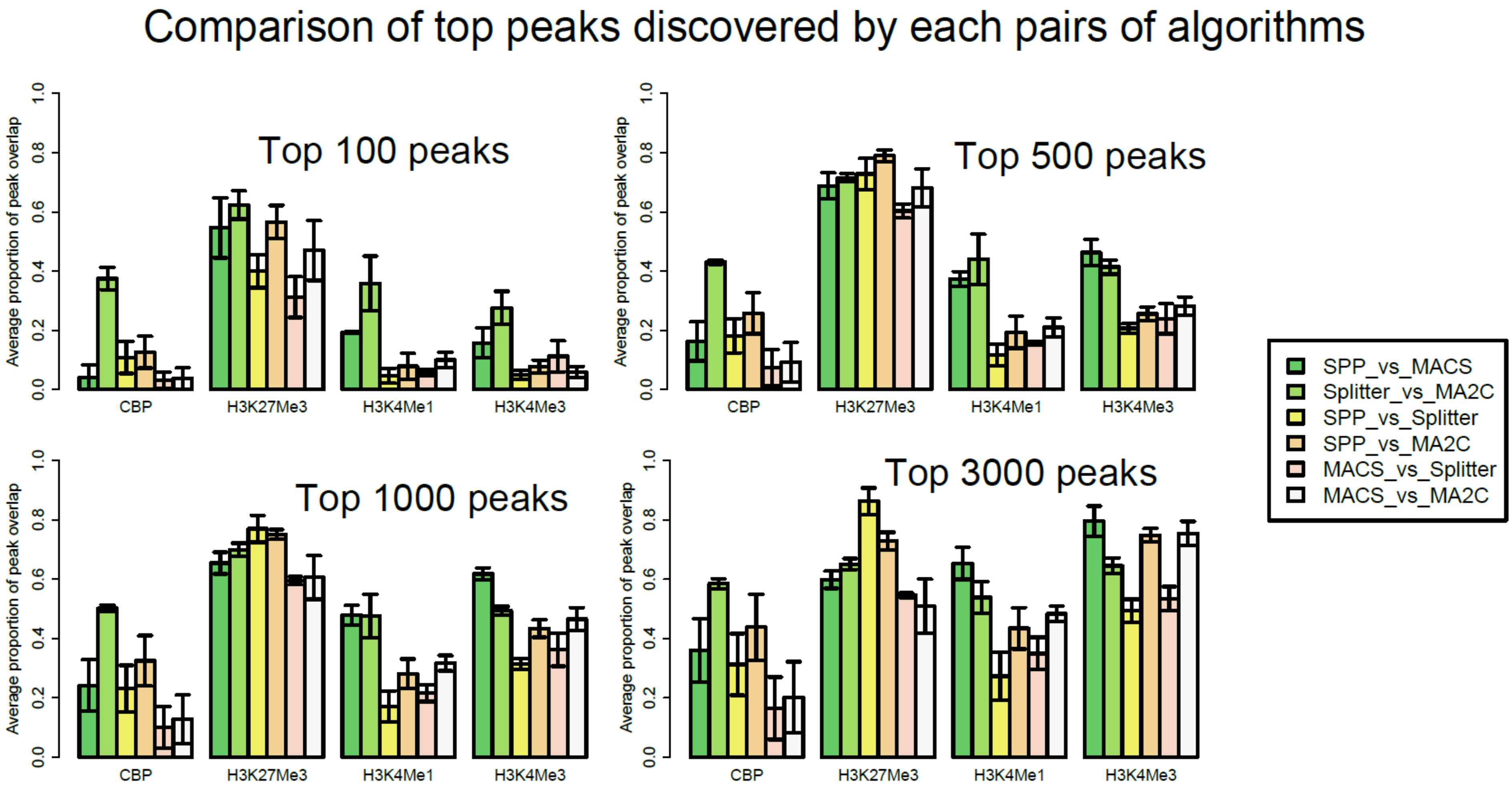

Figure S11. Average proportion of overlap between different number of top *n* peaks (*n*=100,500,3000).
